# Seasonal stoichiometry of terrestrial consumer–resource interactions

**DOI:** 10.1002/ecy.70383

**Published:** 2026-04-09

**Authors:** Richard E. Feldman, Anna Singh, Paul C. Frost

**Affiliations:** ^1^ Wildlife Research and Monitoring Section Ontario Ministry of Natural Resources Peterborough Ontario Canada; ^2^ Department of Biology Trent University Peterborough Ontario Canada

**Keywords:** alvar, Eastern Whip‐poor‐will, foraging, lepidoptera, moths, nitrogen limitation, phenology, seasonality

## Abstract

Variation in producer stoichiometry influences animal distribution, abundance, and behavior. While spatial variation in producer stoichiometry is widely acknowledged, seasonal variation in producer nutrient content may also strongly affect primary consumers. For many terrestrial ecosystems, nitrogen concentration in leaves declines over the growing season and is associated with increasing carbon:nitrogen (C:N) ratios. As a result, late summer folivores like lepidoptera encounter a very different stoichiometric landscape than that of early summer. Although arthropods are assumed to regulate their elemental composition (to some degree), it is possible that the stoichiometry of these communities also varies seasonally in concert with their resources. To quantify the effects of seasonal changes in foliar stoichiometry on insects, we measured carbon and nitrogen concentrations and C:N ratios in leaves and adult moths in an alvar ecosystem of Ontario, Canada, nearly every week from early May to early October 2023. We found that leaf N concentrations decreased, C concentrations remained stable, and C:N ratios increased in leaves across the growing season. The result was consistent across different light levels though somewhat variable across sampling locations. On the contrary, moth N and C concentrations and C:N ratios varied minimally across the growing season. Furthermore, we found little evidence for consistent seasonal declines in per‐individual moth body mass or total moth abundance, and no strong direct association between these moth characteristics and foliar C:N. As a result, there may be a limited physiological cost to acute N limitation in an N scarce landscape. Just how late summer foliage eating arthropods have adapted to high C:N ratios remains a key question, especially since climate change may cause earlier leaf emergence and lengthen periods of consumer–resource stoichiometric mismatch.

## INTRODUCTION

Animals require a mixture of energy and elements for metabolism that may not be met in nutrient‐poor environments (Frost et al., 2005; Raubenheimer et al., 2009). Within the macro‐ and micronutrients that animals consume, nitrogen (N) is a key element that is used in a variety of enzymatic processes and to build structural proteins (Mattson, 1980; Sterner & Elser, 2002). As the concentration of N in the tissues of autotrophs can be an order of magnitude lower than that of heterotrophs (Elser et al., 2000; Mattson, 1980), N limitation can strongly affect herbivore behavior, demography, distribution, and evolution (Fagan et al., 2002; McArt et al., 2009; Rizzuto et al., 2021). Primary consumers should have traits that allow them to overcome elemental deficiencies in producers (Lee et al., 2012; Rosenblatt, 2018) and maintain elemental homeostasis in different environments. On the other hand, there can be interspecific differences in carbon:nitrogen (C:N) ratios (Elser et al., 2000; Fagan et al., 2002) and homeostatic strengths (Persson et al., 2010) among primary consumers like terrestrial arthropods. Thus, turnover in arthropod species across space and time (sensu Murakami et al., 2008) could lead to widely divergent C:N ratios in herbivore prey, making the foraging environment of secondary consumers more heterogenous than widely assumed.

In terrestrial systems, producer C:N ratios vary over broad spatial scales due to abiotic environmental variation (Leroux et al., 2017; Sardans et al., 2011). At finer scales, there is also a commonly seen pattern of foliar N declining through the growing season (Fajardo & Siefert, 2016; Sanders‐DeMott et al., 2018). For deciduous plants, digestible N begins to accumulate as leaves start growing (García‐González et al., 2016), but then is reduced over time as the element is translocated to indigestible fibers (Barboza et al., 2018) and roots (Walsh et al., 1997). If variation in foliar N content among plants over time leads to variation in herbivore nitrogen content (Fagan et al., 2002), then the quality of primary consumers as a resource for secondary consumers could likewise diminish. Alternatively, N limitation could reduce growth and reproduction of homeostatic primary consumers and lead to reduced food quantity for secondary consumers (Ritchie, 2000; White, 1984). The effects of changing leaf N content could become more severe if climate change causes foliar nitrogen to decline earlier in the season (Walsh et al., 1997) producing phenological mismatches between nutritional supplies and consumer demands (Shipley et al., 2022; Twining et al., 2022). Here we use a nutritional approach that recognizes that food quality may be as variable and consequential to consumers as resource quantity (Frost et al., 2005; Razeng & Watson, 2015; Rosenblatt & Schmitz, 2016).

For many bird species, arthropods are a key nitrogen source for both adults and developing young (Boag, 1987; Levey & Karasov, 1989; Reynolds et al., 2003; Tsahar et al., 2006). Larval and adult lepidoptera are a consistently preferred prey type (Moorman et al., 2007; Rytkönen et al., 2019; Wheelwright, 1986), and some birds, notably nocturnal insectivores, may be entirely dependent on adult lepidoptera, that is, moths (English et al., 2017; Sierro et al., 2001). Larval lepidoptera—caterpillars—may be constrained in their ability to compensate for nitrogen shortfalls in the leaves they consume (Lee et al., 2002, 2004). Low leaf N can reduce N content of caterpillars (Lee et al., 2002) and limit individual growth and survival rates (Despland & Noseworthy, 2006). Moreover, poor and elementally imbalanced diets for caterpillars can affect adult populations by delaying metamorphosis (Lee et al., 2012) and reducing adult body mass (Boggs & Freeman, 2005). Birds foraging on N‐limited lepidopteran prey may need to increase foraging activity to compensate for lower moth body size and abundance (sensu Suzuki‐Ohno et al., 2012). However, little is known about seasonal and spatial variation in C:N ratios at the basal levels of the trophic chain leading to a secondary insectivorous consumer. Here we use the Eastern Whip‐poor‐will (*Antrostomus vociferus*), a nocturnal aerial insectivore that forages almost exclusively on adult moths (Souza‐Cole et al., 2022) as a tractable system to understand variation in plant C:N and its potential effects on the insect–bird food chain. Our study occurred in an alvar ecosystem—an open calcareous grassland—of southern Ontario, Canada, where we expected foliar C:N ratios to increase over the course of the summer and to be higher in well‐lit plants growing in the open alvar compared to plants growing in shadier conditions (shaded leaves tend to be rich in nitrogen because they have more pigments, enzymes, and proteins for photosynthesis (Seemann et al., 1987, Xie et al., 2018)). Subsequently, we predicted that N‐limited food available to late season moths would be associated with higher moth C:N ratios. Alternatively, moths may regulate nitrogen leading to C:N ratios that vary little over time and space. However, if nitrogen limitation in food is not compensated through increased feeding or diet choice then we might expect to find seasonal decreases in individual moth body mass and total moth abundance, and more directly, inverse relationships between foliar C:N and moth body mass and abundance.

## METHODS

### Field sampling

**FIGURE 1:**
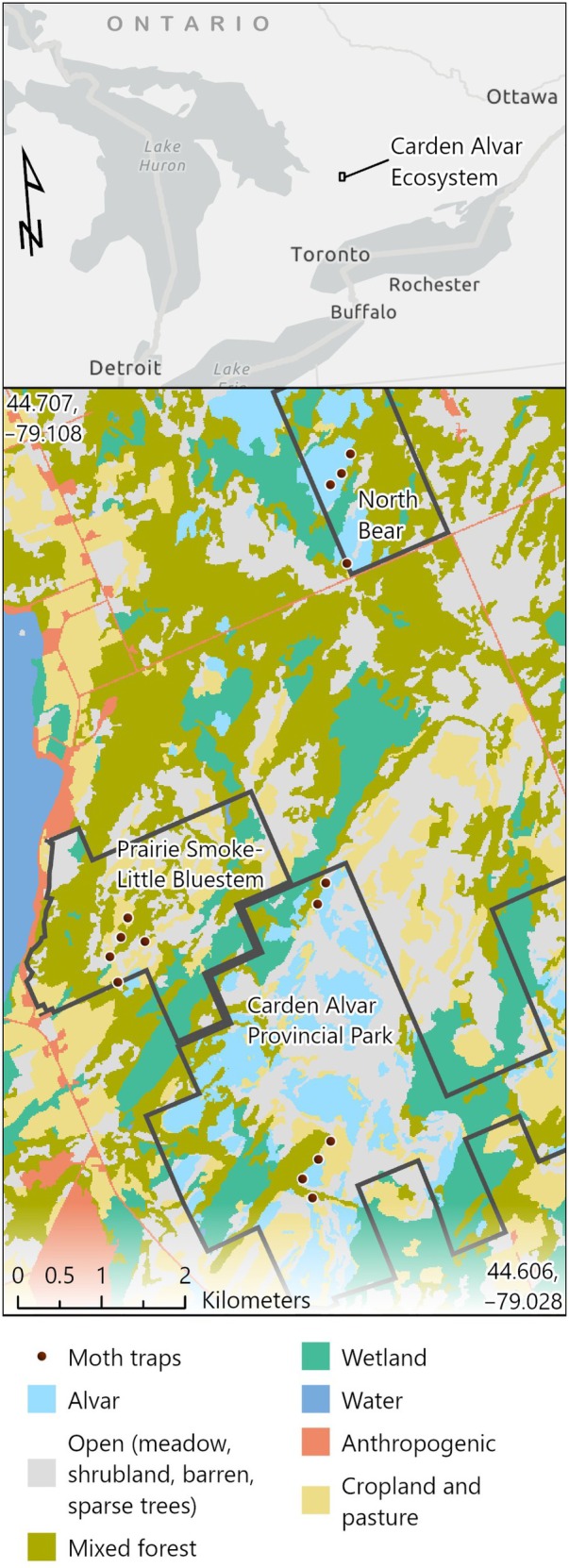
Moth sampling locations in three properties (black borders) within the Carden Alvar region of southern Ontario, Canada, in 2023. Data sources: Habitat—combined classes from Ontario Ministry of Natural Resources Ontario Land Cover Version 1.0 (2023); extent map—Province of Ontario, Esri, TomTom, Garmin, FAO, NOAA, USGS, EPA, NRCan, Parks Canada.

We carried out our study in the Carden Alvar region of southern Ontario, Canada (Figure 1). The landscape is a mix of alvar, mixed forest, cropland, and pastureland. Alvar is open habitat of mostly native grasses (e.g., *Schizachyrium scoparium*) and forbs (e.g., *Geum triflorum*) growing on a thin soil layer set over limestone. There are scattered Common Hawthorn (*Crataegus monogyna*), Common Buckthorn (*Rhamnus cathartica*), Glossy False (Alder) Buckthorn (*Frangula alnus*), and Fragrant Sumac (*Rhus aromatica*) trees growing in the alvar and Common Juniper (*Juniperus communis*) growing near the forest edge. The forests are mostly mixes of Eastern White Cedar (*Thuja occidentalis*), Eastern White Pine (*Pinus strobus*), and Trembling Aspen (*Populus tremuloides*). The juxtaposition of forest for nesting and open alvar for foraging is highly suitable for Eastern Whip‐poor‐will (English et al., 2017; Grahame et al., 2021).

We sampled moths and leaves at 15 randomly placed points along the forest‐alvar edge clustered in three accessible land parcels: Carden Alvar Provincial Park (six points), North Bear (four points), and Prairie Smoke‐Little Bluestem (five points) Nature Conservancy of Canada properties (Figure 1). (Initially, we had a fifth point at North Bear but ceased sampling after 3 weeks due to the time required to access the point.) Within a parcel, the points were separated by a minimum distance between 269.9 and 1340 m.

At each point, we placed a moth trap consisting of a 19‐L plastic bucket and a fluorescent UV bulb (Leptraps Inc.). We powered the trap with a 12‐V battery. Inside the trap, we placed two 118 ml tin cans with sponges soaked in ethyl acetate, which we used as a killing agent. We placed the traps in open alvar. Using the Ontario Land Cover database (Ontario Ministry of Natural Resources, 2023) to define the forest‐alvar border, our traps ended up being 3.15–153 m from the forest edge. From the moth traps, we chose the closest tree or shrub along the forest edge and another individual in the alvar; we marked each plant with flagging tape. We assessed edge and alvar habitats visually in the field, though the distinction is complicated by tree and shrub encroachment. Following land cover delineated by the Ontario Land Cover database (Ontario Ministry of Natural Resources, 2023), our edge plants were 0.00–142 m from a forest edge and our alvar plants were 18.1–227 m from the forest edge. Our most frequently sampled plant was Trembling Aspen, followed by Chokecherry (*Prunus virginiana*) and Fireberry Hawthorn (*Crataegus chrysocarpa*) with eight additional species that we sampled at one or two points.

We collected data 10–17 times from May 6 to October 6, 2023. On average, we sampled leaves every 10.4 ± 5.55 days and moths every 10.7 ± 5.75 days. On each visit to a marked plant, we randomly sampled three leaves from breast height. Above each leaf, we placed a cell phone with the Photone light meter application (Lightray Innovation GmbH) installed and running. We set the application to measure the daily light interval over a 12‐h period, which we later converted from mol E per square meter per day to photosynthetic photon flux density (in micromoles E per second per square meter).

To sample the moths, we used a timer that switches on as light diminishes in the evening and then switches off as light increases at dawn. We could not be sure of the exact duration over which the fluorescent bulb was running. Of 229 potential moth samples, 30 times we collected only one or zero moths, due to inclement weather or a malfunctioning trap. On 67 occasions, we collected so many moths that we subsampled 33–62 randomly chosen individuals to bring back to the lab for elemental analysis. Otherwise, we collected the whole sample.

### Measuring moth and leaf elemental composition

Once the leaves and moths were in the lab, we weighed the samples, placed them in whirl‐pak bags and freeze‐dried them at −50°C for 48 h. We stored the freeze‐dried samples in a −20°C freezer for 1–4 months, after which we homogenized the samples, initially using a mortar and pestle for leaves and a coffee grinder for moths. We then homogenized the samples further using a bead homogenizer (Standard BBX24) until each sample was a fine powder. We transferred the homogenized samples into glass vials.

We subsampled 3.0–5.0 mg of each leaf sample and 2.0–3.0 mg of each moth sample. We analyzed C and N content of each subsample using a Vario EL Cube elemental analyzer (Elementar Analysensysteme GmbH). As a control, we used BOVM‐1 prepared from Canada Grade A beef muscle. We report all ratios as molar ratios.

For our analyses, we used raw C:N ratios rather than the natural logarithm (sensu Isles, 2020) to more clearly compare our results to other studies. We modeled ratios using a Gamma distribution and log link, which means that we relate the log of the expected ratio to our linear predictors.

### Analysis

We estimated the linear week‐by‐week change in %N, %C, and C:N for leaves taken from plants on the forest edge and in the alvar, and for moths. We modeled N and C composition as proportions, and therefore, drawn from a Beta distribution because 0 < N, C < 1. Meanwhile, we modeled C:N as drawn from a gamma distribution because 0 < C:N. For both distributions we modeled the mean (beta distribution) and rate (gamma distribution) as a function of the sample type (edge leaf, alvar leaf, moth) and the interaction between sample type and sample week, meaning each sample type could have a different intercept and slope (change through time). Because elemental composition is repeatedly measured from the same point over time, point specific parameter estimates (i.e., random effects) were drawn from a multivariate normal distribution. We rescaled sampling weeks so they fell within a range of 0–1.

Given that “alvar” and “edge” may be weakly informative of leaf growing conditions, we estimated directly the effect of light availability on leaf %N, %C, and C:N. Because light levels can vary with time of day, cloud cover, and season, we averaged the photosynthetic photon flux density values taken (nearly) weekly for each leaf sample to provide the sample plant's general light conditions. We used beta and Gaussian error distributions for the elemental proportions and ratios, respectively. We modeled mean as a function of light (photosynthetic photon flux density). With the same plant sampled over multiple weeks and the same weeks covering multiple plants, we included plant and time random effects that were drawn from normal distributions.

We tested for seasonal declines in individual and assemblage level moth attributes by estimating temporal variation in per‐individual moth body mass and total moth abundance. We obtained per‐individual moth body mass by dividing total biomass of each trap's sample by the number of moths in the sample. For total abundance, we often stopped counting when there were more than approximately 50 moths in the trap. Consequently, we set the values of those traps at 50 and we accounted for the right censoring in our model (Stan Development Team, 2024). We modeled mean per‐individual body mass with a lognormal distribution and total abundance with a Gaussian distribution and we related the means to a smoothed outcome of sampling week that we rescaled to lie between 0 and 1. We used smoothing (i.e., generalized additive modeling) because early and late season moth characteristics could be similar. We also included a factor smooth of the interaction between sampling week and moth trap, which is equivalent to the shape of the smoothed temporal effect randomly varying across traps but sharing the same “wigiliness” (Pedersen et al., 2019). We set the number of basis functions for the smooths, *k*, to five. We added foliar C:N (averaged between alvar and edge leaves and then scaled) as a predictor to test whether there was any notable effect of leaf elemental composition on moths above and beyond the seasonal relationships.

To the moth body mass and abundance models, we added trap as a random effect. Furthermore, we adjusted the week and ratio effects by including temperature, wind speed, and moonlight intensity as scaled covariates. We accessed weather data from Environment and Climate Change Canada using the weathercan package (LaZerte & Albers, 2018) in R (R Development Core Team, 2024). We downloaded hourly data that we clipped to sunset and sunrise for each trap night and averaged temperature and wind speed over the night. We accessed moonlight intensity values for each trap night using the moonlit package (Śmielak, 2024) in R (R Development Core Team, 2024). Moonlight intensity is calculated from a model that accounts for moon phase, distance from the moon, and moon visibility (Śmielak, 2023).

We ran all models using the brms v. 2.21.0 package (Bürkner, 2017, 2018, 2021) in R v. 4.4.2 (R Development Core Team, 2024) running cmdstanr v. 0.8.0 (Gabry et al., 2024) as the interface to Stan v. 2.35 (Stan Development Team, 2024) where the models were compiled and run. We chose the priors to be weakly informative, resulting in parameter estimates with few extreme values. For C:N, the priors, when back‐transformed, produce a distribution of C:N centered on 1:1. All statistical models are detailed in Appendix S1. We used Microsoft Copilot to help write the correct statistical notation; however, coding and running the models were all done by the authors. Data, R code, and prior and posterior predictive checks are available at (Feldman, 2026; https://doi.org/10.5281/zenodo.18749747).

## RESULTS

### Leaf and moth elemental composition and ratios through time

**FIGURE 2:**
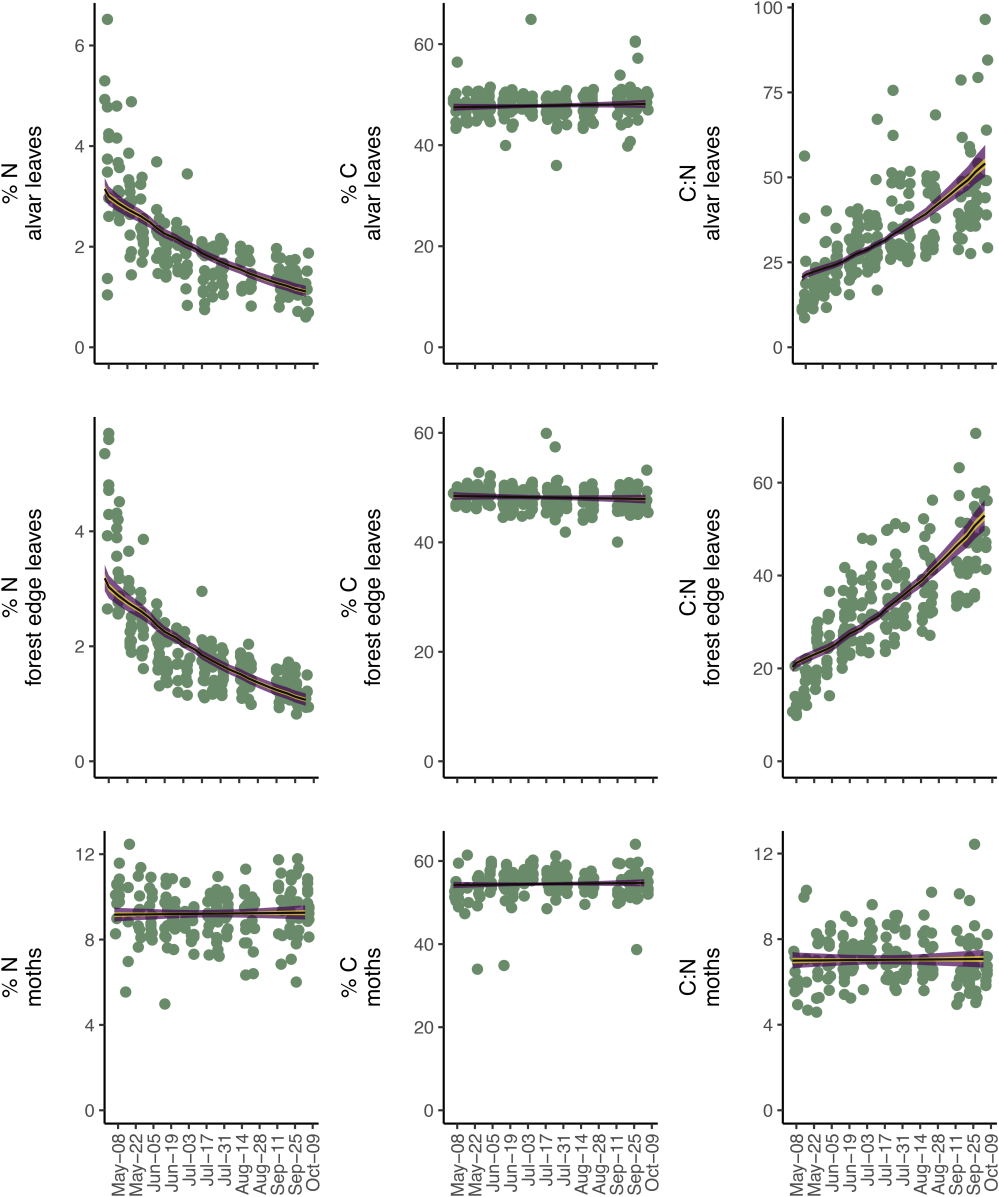
Seasonal change in carbon and nitrogen content and C:N ratios for leaves taken from plants growing in the open alvar and forest edge and from moths. The yellow and purple shadings represent the 50% and 95% credible intervals of the posterior distributions. The black lines are the medians of the posterior distributions. The green points are the observed contents and ratios for the leaf and moth samples from each of 15 sampling points at different time points across the season.

At the start of the season, on May 6, 2023, moths contained proportionally more carbon and nitrogen, and lower molar C:N ratios than leaves collected along the forest edge or in the open alvar (Figure 2). C:N ratios were similar between edge and alvar leaves (Figure 2), and we found essentially no variation in nitrogen content (slope and 95% credible interval: −0.0238 [−0.0569, 0.00920]), carbon content (slope and 95% credible interval: −0.0167 [−0.168, 0.138]), and C:N ratio (slope and 95% credible interval: 0.00599 [0.00133, 0.0107]) with increasing light levels (100 μmol E/s m^2^) (Appendix S2: Figure S1).

Over the course of the growing season, carbon and nitrogen content and C:N ratios were stable in moths as was carbon content in leaves (Table 1; Figure 2). However, there was a notable decline in leaf nitrogen content, leading to increasing C:N ratios (Table 1; Figure 2). (Given the appearance of a possible nonlinear seasonal patterns in the raw elemental composition and ratio data, we explored generalized additive models to account for smoothed relationships with sample weeks. The models revealed that foliar nitrogen declines most steeply at the start of the summer and that there might be a modest unimodal hump in seasonal moth C:N ratios, trending from 6.31 [5.72, 6.96] to a peak at 7.43 [7.08, 7.81] and ending at 6.90 [6.36, 7.49] (Appendix S3). With such a negligible change, we carry on interpreting the linear model).

**TABLE 1:** The predicted change in elemental composition and ratios between May 6 and October 6 for leaves and moths in the Carden Alvar ecosystem. The predictions are averaged across the study sites.

Element	Taxa	Seasonal change, median, and 95% credible interval	SD of random effects describing among site variation, median, and 95% credible interval^a^	
			Intercept	Slope
%C	Alvar leaves	0.661 [−0.621, 1.95]	1.08 [1.04, 1.13]	1.03 [1, 1.12]
	Edge leaves	−0.584 [−1.91, 0.729]	1.04 [1.01, 1.08]	1.03 [1, 1.11]
	Moths	0.477 [−0.906, 1.9]	1.11 [1.06, 1.18]	1.18 [1.1, 1.3]
%N	Alvar leaves	−1.97 [−2.23, −1.72]	1.19 [1.11, 1.35]	1.13 [1.01, 1.46]
	Edge leaves	−2.04 [−2.31, −1.78]	1.12 [1.03, 1.23]	1.07 [1, 1.27]
	Moths	0.0955 [−0.474, 0.673]	1.01 [1, 1.05]	1.02 [1, 1.09]
C:N	Alvar leaves	31.5 [27.6, 35.8]	1.28 [1.18, 1.47]	1.49 [1.21, 2.21]
	Edge leaves	31.1 [27.6, 34.6]	1.16 [1.09, 1.28]	1.06 [1, 1.23]
	Moths	0.073 [−0.599, 0.758]	1.01 [1, 1.05]	1.03 [1, 1.12]

^a^ The SD of the normal distribution for the hyperparameters of site level intercepts (at the start of the sampling season) and slopes (change in elemental composition and ratios over the course of the sampling season) for carbon and nitrogen composition and C:N ratios for leaves taken from plants growing in the open alvar and forest edge and from moths. The values have been exponentiated to the response scale.

Leaf nitrogen content, C:N ratios, and their change through the season varied spatially, that is, across the 15 sampling points, and more so for leaves taken from the open alvar than from the edge or for moths (Table 1; Appendix S2: Figures S2 and S3). Meanwhile, spatial variation in carbon content and its change over time was more variable for moths than for leaves (Table 1; Appendix S2: Figures S2 and S3).

### Per‐individual moth body mass and total abundance

**FIGURE 3:**
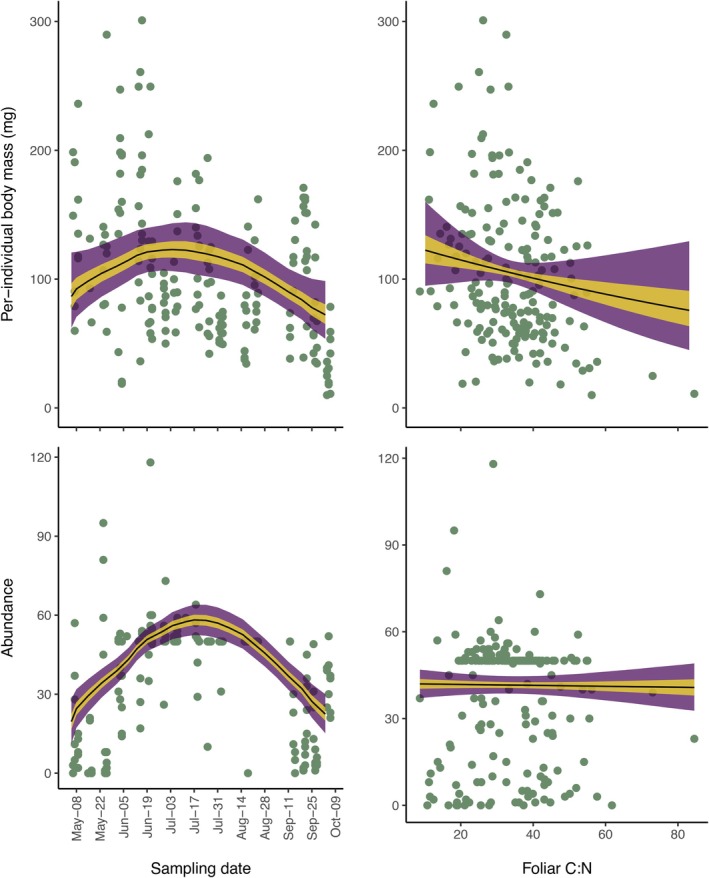
The seasonal change in per‐individual moth body mass and total moth abundance per capture night and the relationship between those same variables and foliar C:N ratios. The yellow and purple shadings represent the 50% and 95% credible intervals of the posterior distributions. The black lines are the medians of the posterior distributions. The green points are the observed body masses and abundances from each of 15 sampling points at different time points across the season.

We found that per‐individual moth body mass exhibited a pronounced seasonal pattern; however, estimates of body mass differences between the beginning and end of the growing season were unclear in their magnitude and direction. On average moths were 80.4 mg [95% credible interval: 62.2, 121] on May 6 and 72.3 mg [95% credible interval: 53.7, 98.5] on October 6 (Figure 3). The seasonal trend was not linear: per‐individual moth body masses increased modestly until July 4 (123 mg [95% credible interval: 106, 143]) and then declined steeply through the rest of the summer. In contrast, total moth abundance peaked a little later (July 17 at 58.2 individuals [52.5, 64.0]) and abundances were similar in May and October (19.6 [11.1, 28.2] vs. 22.4 [15.2, 30.0]) (Figure 3). After accounting for the seasonal pattern in body mass and temperature, windspeed, and moonlight intensity, we found a weak and variable relationship with foliar C:N ratio (Table 2; Figure 3). Similarly, there was no clear relationship between total abundance and foliar C:N (Table 2; Figure 3). There was very little among‐trap variation in per‐individual body mass and total abundance (Table 2; Appendix S2: Figure S4).

**TABLE 2:** The predicted change in per‐individual moth body mass and total moth abundance with an increase in foliar C:N ratios from 34:1 to 45:1 in the Carden Alvar ecosystem. The predictions are averaged across the study sites.

Moth property	Change with one SD increase in C:N, median, and 95% credible interval	SD of random effects describing among site variation, median, and 95% credible interval^a^
Per‐individual body mass	−1.13 (−9.76, 1.59)	43.7 (35.0, 53.5)
Total abundance	0.091 (−2.12, 2.26)	25.3 (20.5, 31.3)

^a^ The SD of the normal distribution for the hyperparameters of site level intercepts (i.e., at covariate means, including C:N = 33.6).

## DISCUSSION

How animals have adapted to spatial and temporal variation in the elemental composition of their food is key for understanding the consequences of global change (McLeod et al., 2025; Sardans et al., 2021). Anthropogenic activities like agricultural intensification and climate change are changing the elemental landscape (Dijkstra et al., 2012), which may then affect animal populations and ecosystem structure and function (Carnicer et al., 2015). Most amazingly is the notion—informed by a body of research—that elemental composition can vary substantially among producers but minimally among consumers (Sterner & Elser, 2002). While the pattern has been demonstrated by pooling data from across taxa, ecosystems, and time periods (e.g., Elser et al., 2000; Persson et al., 2010), measuring elemental variation within a(n) (meta‐) ecosystem is key for quantifying spatial and temporal variation in elemental imbalances between trophic levels. In our study, we collected stoichiometric data for plants and moths in an alvar ecosystem of southern Ontario, Canada. Our data and analyses support the general phenomenon: we found that foliar C:N ratios increased through the growing season while moth ratios stayed nearly constant.

In our ecosystem, producers were shrubs and small trees growing mostly unshaded along or near the edge between forest and alvar, an open calcareous grassland. One of our main findings was that molar C:N ratios in leaves increased across the growing season from approximately 20:1 in early May to a little over 50:1 in early October. Further, we found that the ratios increased because nitrogen concentration declined from approximately 3.1% to 1.1%, but carbon concentration remained steady at 48%. We found that the trends were roughly the same regardless of the light conditions within which the plant was growing, though there was some variation based on the spatial location of the plant. Our ratios correspond closely to terrestrial producers measured across the globe (Elser et al., 2000). Our finding of seasonal foliar nitrogen decline is a characteristic common to many plants (Scriber & Slansky, 1981), including for tree species in eastern North America (Hunter & Lechowicz, 1992) and herbaceous vegetation in North American grasslands (Kaspari & Welti, 2023). The decline corresponds to physiological changes in plants over the growing season: as leaves mature and senesce, nitrogen is translocated to and resorbed by other tissues (Aerts, 1996; Aerts & Chapin, 2000; Brant & Chen, 2015). As a consequence, herbivores may be especially nitrogen limited in late summer (Barboza et al., 2018; McArt et al., 2009).

Despite the declining nitrogen and increasing C:N ratios in leaves, we found that the herbivores we measured—moths—had elemental compositions and ratios that varied minimally over the growing season. Our finding of 9% nitrogen is consistent with a broad survey of published and unpublished data on adult lepidoptera (Fagan et al., 2002). Likewise, our finding of C:N ratios of approximately 7:1 is consistent with terrestrial arthropods (Elser et al., 2000). Although our results are consistent with strong stoichiometric regulation of body N content, we caution against inferring strict homeostasis from these data. We do not have data from caterpillars, the life phase that consumes leaves, nor do we know the host plants upon which our moths forage. Without data on the individuals involved in the consumer–resource interaction, we are limited to describing the general nutrient content of key players in the alvar ecosystem. While larval lepidoptera tend to have lower C:N ratios than adults (Fagan et al., 2002; Studier et al., 1991), many moths do not feed as adults, so the temporal patterns in nutrient composition may be the same in both life stages. Alternatively, it is possible that nitrogen declines in larvae, to which adults compensate by consuming pollen (Wäckers et al., 2007).

One of the key implications of our study is that late season lepidoptera are foraging in a more nitrogen‐limited landscape than their early‐season counterparts. If late season moths are compensating for nitrogen scarcity through behavioral or physiological adaptations, then we might expect to see seasonal variation in individual phenotype that reflects the costs of expressing such adaptations. Within a species, moth caterpillars foraging on nitrogen deficient food later in the summer grow slower than those individuals foraging earlier in the summer (Hunter & Lechowicz, 1992). Likewise, feeding trials have found that caterpillars foraging on low nitrogen diets have reduced growth (Despland & Noseworthy, 2006), and lower tissue protein content (Lee et al., 2012) than moths fed balanced diets. The negative outcomes to caterpillars can result in lower adult body mass and wing length (Boggs & Freeman, 2005). In our study, we did not find convincing evidence of a physiological cost to N limitation; posterior distributions of seasonal differences in per‐individual moth body mass and its association with foliar C:N were broad, with considerable mass above and below zero. Therefore, increases, decreases, or no effect are all plausible interpretations given the data. It is possible that we failed to detect a strong change in body mass because we never sampled micromoths (<10 mm). We did not track how many micromoths we discarded each night, and it is possible that we overestimated body mass on some sampling nights. In addition, we measured fresh biomass, so the presence of particularly humid nights could obscure body mass trends. Nonetheless, our findings reveal that late season lepidoptera may be adapted to low nitrogen content leaves. While there are some studies of interspecific differences in the capacity to compensate for nutrient imbalances in terrestrial insects (Lee et al., 2003; Raubenheimer & Simpson, 2003), there has yet to be any comparison of nitrogen regulation among the species present at different points of the seasonal foliar nitrogen decline.

Irrespective of the effects on individual moth traits, nitrogen limitation might also be manifest at the population level, that is, lower abundances (sensu Ritchie, 2000), especially given that caterpillars foraging on low nitrogen diets have lower survival (Lindroth et al., 1997; Raubenheimer et al., 2005). However, estimates of abundance change between the beginning and end of the season or between low and high foliar C:N were small and imprecise. More pronounced were mid‐summer declines in abundance—and per‐individual moth body mass—following seasonal peaks. Hence, we cannot rule out that nitrogen limitation affected moths only at the end of the season, and that earlier in the year other elements, possibly phosphorus, were more limiting and required a greater degree of regulation than nitrogen (Elser et al., 2000, Lemoine et al., 2014, but see Zhu et al., 2021). Phosphorus may also vary more consistently with environmental conditions than nitrogen (Dynarski et al. 2023) and may be why we measured so little spatial variation in the seasonal trends of moth C:N. Even more likely is that moth abundance reflects the combination of multiple nutrients available in foliar tissue, including potassium and micronutrients like sodium (Prather et al., 2020). Our sample sites at Carden Alvar Provincial Park are grazed by cattle that deposit potassium and sodium into the soil via urine. Potassium and sodium fertilization can suppress insect abundances when nitrogen is high but lead to increasing insect abundance as nitrogen declines (Kaspari & Welti, 2023). On the other hand, we did not find that our ungrazed sites (Prairie Smoke and North Bear) had consistently different body masses or abundances than grazed sites (Carden Alvar Provincial Park).

Foliar nitrogen and C:N ratios vary intra‐ and interspecifically depending on species traits and the local biotic and abiotic environment (Borer et al., 2015; Dynarski et al., 2023; Feng et al., 2024; Lu et al., 2023; Sterner & Elser, 2002). On theoretical grounds, higher light levels should reduce foliar nitrogen and increase C:N ratios because plants fix more carbon and require less nitrogen in their photosynthetic pigments (Cronin & Lodge, 2003; Evans, 1989). In practice, light is confounded with soil elemental composition, soil moisture, and temperature (Kranabetter & Simard, 2008; Sun et al., 2019). Nonetheless, we designed our study to measure foliar nitrogen and carbon in plants growing in shade and sunlight, which resulted in sampling seven plants between 150 and 280 μmol E/s m^2^ and 34 plants between 400 and 900 μmol E/s m^2^. Across the light gradient, we found a weak and sign‐varying relationship with carbon and nitrogen content and C:N ratios. We may not have found a convincing relationship with light because we did not sample a wide enough range of light conditions or, perhaps, our cell phone application and measure of photon flux density did not accurately capture solar irradiance. While we did not find any convincing relationship with light, we found spatial stoichiometric variation among individual plants, which could reflect edaphic features uncorrelated with light and the characterization of alvar and edge habitats. Whether soil nitrogen availability is one of them is questionable since we did not find consistent stoichiometric differences between our grazed and ungrazed sites. Previous studies have found that grazing alters soil nitrogen availability and foliar nitrogen content compared to ungrazed systems (Hassan et al., 2021; Sitters et al., 2017).

In our alvar ecosystem, we found strong evidence that moths regulate nitrogen such that individuals flying at different points during the growing season have the same C:N ratios, despite a marked increase in the ratios in their food. Future studies could interrogate two alternative hypotheses about how late season lepidoptera regulate nitrogen. One possibility is that late season moths are more specialized than their early‐season counterparts and overcome nutrient imbalances by more efficiently converting nitrogen to growth (Behmer, 2009). Another is that late season moths are more generalist because they can mix elementally imbalanced woody vegetation tissue with more balanced herbaceous vegetation (sensu Raubenheimer & Simpson, 2003, Simpson et al., 2004). The generalist hypothesis is predicated on the herbaceous layer renewing itself with new growth over the summer, and thus, providing consistent C:N ratios, even if nitrogen concentration, itself, is lower than in woody vegetation. Furthermore, we cannot rule out that changes in the moth assemblage and individual phenotype are as much a response to spatiotemporal variation in leaf phosphorus and N:P ratios or other micro and macronutrients as C:N. A study of seasonal nutrient variation could be extended to consider C:N:P ratios and even the whole spectrum of elements (sensu Frost et al., 2005). Consequently, a way forward is to quantify the full stochiometric niche (sensu González et al., 2017) and discover the mechanisms driving patterns of niche divergence or convergence between producers and consumers over the growing season. If climate change induces or exacerbates niche differences, then there may be effects that propagate through the food web (Diehl et al., 2022; Zhang et al., 2022). In an alvar ecosystem like ours that is home to an at‐risk insectivorous bird species, the Eastern Whip‐poor‐will, the future of the quantity and quality of their food could be shaped by how moths regulate their nutrient composition in the face of drastic changes in the stoichiometry of the leaves they consume.

## AUTHOR CONTRIBUTIONS

Richard E. Feldman conceived and designed the research. Anna Singh and Paul C. Frost processed and analyzed the samples and Anna Singh and Richard E. Feldman conducted the statistical analyses. All authors contributed to writing and revising the manuscript.

## CONFLICT OF INTEREST STATEMENT

The authors declare no conflict of interest.

## Supporting information


Appendix S1.

Appendix S2.

Appendix S3.

## Data Availability

Data, code, and model diagnostics (Feldman, 2026) are available in Zenodo at https://doi.org/10.5281/zenodo.18749747.

## References

[ecy70383-bib-0001] Aerts, R. 1996. “Nutrient Resorption from Senescing Leaves of Perennials: Are there General Patterns?” The Journal of Ecology 84: 597.

[ecy70383-bib-0002] Aerts, R. , and F. S. Chapin . 2000. “The Mineral Nutrition of Wild Plants Revisited: A Re‐Evaluation of Processes and Patterns.” Advances in Ecological Research 30: 1–67.

[ecy70383-bib-0003] Barboza, P. S. , L. L. Van Someren , D. D. Gustine , and M. S. Bret‐Harte . 2018. “The Nitrogen Window for Arctic Herbivores: Plant Phenology and Protein Gain of Migratory Caribou (*Rangifer tarandus*).” Ecosphere 9: e02073.

[ecy70383-bib-0004] Behmer, S. T. 2009. “Insect Herbivore Nutrient Regulation.” Annual Review of Entomology 54: 165–187. 10.1146/annurev.ento.54.110807.090537 18764740

[ecy70383-bib-0005] Boag, P. T. 1987. “Effects of Nestling Diet on Growth and Adult Size of Zebra Finches (*Poephila guttata*).” The Auk 104: 155–166.

[ecy70383-bib-0006] Boggs, C. L. , and K. D. Freeman . 2005. “Larval Food Limitation in Butterflies: Effects on Adult Resource Allocation and Fitness.” Oecologia 144: 353–361. 15891831 10.1007/s00442-005-0076-6

[ecy70383-bib-0007] Borer, E. T. , E. M. Lind , E. J. Ogdahl , E. W. Seabloom , D. Tilman , R. A. Montgomery , and L. L. Kinkel . 2015. “Food‐Web Composition and Plant Diversity Control Foliar Nutrient Content and Stoichiometry.” Journal of Ecology 103: 1432–1441.

[ecy70383-bib-0008] Brant, A. N. , and H. Y. H. Chen . 2015. “Patterns and Mechanisms of Nutrient Resorption in Plants.” Critical Reviews in Plant Sciences 34: 471–486.

[ecy70383-bib-0009] Bürkner, P.‐C. 2017. “brms: An R Package for Bayesian Multilevel Models Using Stan.” Journal of Statistical Software 80: 1–28.

[ecy70383-bib-0010] Bürkner, P.‐C. 2018. “Advanced Bayesian Multilevel Modeling with the R Package brms.” The R Journal 10: 395–411.

[ecy70383-bib-0011] Bürkner, P.‐C. 2021. “Bayesian Item Response Modeling in R with brms and Stan.” Journal of Statistical Software 100: 1–54.

[ecy70383-bib-0012] Carnicer, J. , J. Sardans , C. Stefanescu , A. Ubach , M. Bartrons , D. Asensio , and J. Peñuelas . 2015. “Global Biodiversity, Stoichiometry and Ecosystem Function Responses to Human‐Induced C–N–P Imbalances.” Journal of Plant Physiology 172: 82–91. 25270104 10.1016/j.jplph.2014.07.022 PMC6485510

[ecy70383-bib-0013] Cronin, G. , and D. M. Lodge . 2003. “Effects of Light and Nutrient Availability on the Growth, Allocation, Carbon/Nitrogen Balance, Phenolic Chemistry, and Resistance to Herbivory of Two Freshwater Macrophytes.” Oecologia 137: 32–41. 12820064 10.1007/s00442-003-1315-3

[ecy70383-bib-0014] Despland, E. , and M. Noseworthy . 2006. “How Well Do Specialist Feeders Regulate Nutrient Intake? Evidence from a Gregarious Tree‐Feeding Caterpillar.” Journal of Experimental Biology 209: 1301–1309. 16547301 10.1242/jeb.02130

[ecy70383-bib-0015] Diehl, S. , S. A. Berger , W. Uszko , and H. Stibor . 2022. “Stoichiometric Mismatch Causes a Warming‐Induced Regime Shift in Experimental Plankton Communities.” Ecology 103: e3674. 35253210 10.1002/ecy.3674 PMC9285514

[ecy70383-bib-0016] Dijkstra, F. A. , E. Pendall , J. A. Morgan , D. M. Blumenthal , Y. Carrillo , D. R. LeCain , R. F. Follett , and D. G. Williams . 2012. “Climate Change Alters Stoichiometry of Phosphorus and Nitrogen in a Semiarid Grassland.” New Phytologist 196: 807–815. 23005343 10.1111/j.1469-8137.2012.04349.x

[ecy70383-bib-0017] Dynarski, K. A. , F. M. Soper , S. C. Reed , W. R. Wieder , and C. C. Cleveland . 2023. “Patterns and Controls of Foliar Nutrient Stoichiometry and Flexibility across United States Forests.” Ecology 104: e3909. 36326547 10.1002/ecy.3909

[ecy70383-bib-0018] Elser, J. J. , W. F. Fagan , R. F. Denno , D. R. Dobberfuhl , A. Folarin , A. Huberty , S. Interlandi , et al. 2000. “Nutritional Constraints in Terrestrial and Freshwater Food Webs.” Nature 408: 578–580. 11117743 10.1038/35046058

[ecy70383-bib-0019] English, P. A. , J. J. Nocera , B. A. Pond , and D. J. Green . 2017. “Habitat and Food Supply Across Multiple Spatial Scales Influence the Distribution and Abundance of a Nocturnal Aerial Insectivore.” Landscape Ecology 32: 343–359.

[ecy70383-bib-0020] Evans, J. R. 1989. “Photosynthesis and Nitrogen Relationships in Leaves of C3 Plants.” Oecologia 78: 9–19. 28311896 10.1007/BF00377192

[ecy70383-bib-0021] Fagan, W. F. , E. Siemann , C. Mitter , R. F. Denno , A. F. Huberty , H. A. Woods , and J. J. Elser . 2002. “Nitrogen in Insects: Implications for Trophic Complexity and Species Diversification.” The American Naturalist 160: 784–802. 10.1086/343879 18707465

[ecy70383-bib-0022] Fajardo, A. , and A. Siefert . 2016. “Phenological Variation of Leaf Functional Traits within Species.” Oecologia 180: 951–959. 26796408 10.1007/s00442-016-3545-1

[ecy70383-bib-0023] Feldman, R. E. 2026. “Data and Code for: Seasonal Stoichiometry of Terrestrial Consumer‐Resource Interactions (v3.0).” Zenodo. 10.5281/zenodo.18749747 PMC13063215 41954089

[ecy70383-bib-0024] Feng, W.‐L. , J.‐L. Yang , L.‐G. Xu , and G.‐L. Zhang . 2024. “The Spatial Variations and Driving Factors of C, N, P Stoichiometric Characteristics of Plant and Soil in the Terrestrial Ecosystem.” Science of the Total Environment 951: 175543. 39153619 10.1016/j.scitotenv.2024.175543

[ecy70383-bib-0025] Frost, P. C. , M. A. Evans‐White , Z. V. Finkel , T. C. Jensen , and V. Matzek . 2005. “Are you What you Eat? Physiological Constraints on Organismal Stoichiometry in an Elementally Imbalanced World.” Oikos 109: 18–28.

[ecy70383-bib-0026] Gabry, J. , R. Češnovar , A. Johnson , and S. Bronder . 2024. “cmdstanr: R Interface to ‘CmdStan.’” R package Version 0.8.1. https://discourse.mc-stan.org, https://mc-stan.org/cmdstanr/.

[ecy70383-bib-0027] García‐González, R. , A. Aldezabal , N. A. Laskurain , A. Margalida , and C. Novoa . 2016. “Influence of Snowmelt Timing on the Diet Quality of Pyrenean Rock Ptarmigan (*Lagopus muta* Pyrenaica): Implications for Reproductive Success.” PLoS One 11: e0148632. 26849356 10.1371/journal.pone.0148632 PMC4746074

[ecy70383-bib-0028] González, A. L. , O. Dézerald , P. A. Marquet , G. Q. Romero , and D. S. Srivastava . 2017. “The Multidimensional Stoichiometric Niche.” Frontiers in Ecology and Evolution 5: 110.

[ecy70383-bib-0029] Grahame, E. R. M. , K. D. Martin , E. A. Gow , and D. R. Norris . 2021. “Diurnal and Nocturnal Habitat Preference of Eastern Whip‐Poor‐Wills (*Antrostomus vociferus*) in the Northern Portion of Their Breeding Range.” Avian Conservation and Ecology 16: art14.

[ecy70383-bib-0030] Hassan, N. , X. Li , J. Wang , H. Zhu , P. Nummi , D. Wang , D. Finke , and Z. Zhong . 2021. “Effects of Grazing on C:N:P Stoichiometry Attenuate from Soils to Plants and Insect Herbivores in a Semi‐Arid Grassland.” Oecologia 195: 785–795. 33616723 10.1007/s00442-021-04873-3

[ecy70383-bib-0031] Hunter, A. F. , and M. J. Lechowicz . 1992. “Foliage Quality Changes During Canopy Development of Some Northern Hardwood Trees.” Oecologia 89: 316–323. 28313079 10.1007/BF00317408

[ecy70383-bib-0032] Isles, P. D. F. 2020. “The Misuse of Ratios in Ecological Stoichiometry.” Ecology 101: e03153. 32731303 10.1002/ecy.3153

[ecy70383-bib-0033] Kaspari, M. , and E. A. R. Welti . 2023. “Electrolytes on the Prairie: How Urine‐Like Additions of Na and K Shape the Dynamics of a Grassland Food Web.” Ecology 104: e3856. 36053835 10.1002/ecy.3856

[ecy70383-bib-0034] Kranabetter, J. M. , and S. W. Simard . 2008. “Inverse Relationship Between Understory Light and Foliar Nitrogen Along Productivity Gradients of Boreal Forests.” Canadian Journal of Forest Research 38: 2487–2496.

[ecy70383-bib-0035] LaZerte, S. Z. , and S. Albers . 2018. “weathercan: Download and Format Data from Environment and Climate Change Canada.” The Journal of Open Source Software 3: 571.

[ecy70383-bib-0036] Lee, K. P. , S. T. Behmer , S. J. Simpson , and D. Raubenheimer . 2002. “A Geometric Analysis of Nutrient Regulation in the Generalist Caterpillar *Spodoptera littoralis* (Boisduval).” Journal of Insect Physiology 48: 655–665. 12770076 10.1016/s0022-1910(02)00088-4

[ecy70383-bib-0037] Lee, K. P. , S.‐T. Kwon , and C. Roh . 2012. “Caterpillars Use Developmental Plasticity and Diet Choice to Overcome the Early Life Experience of Nutritional Imbalance.” Animal Behaviour 84: 785–793.

[ecy70383-bib-0038] Lee, K. P. , D. Raubenheimer , S. T. Behmer , and S. J. Simpson . 2003. “A Correlation between Macronutrient Balancing and Insect Host‐Plant Range: Evidence from the Specialist Caterpillar *Spodoptera exempta* (Walker).” Journal of Insect Physiology 49: 1161–1171. 14624888 10.1016/j.jinsphys.2003.08.013

[ecy70383-bib-0039] Lee, K. P. , D. Raubenheimer , and S. J. Simpson . 2004. “The Effects of Nutritional Imbalance on Compensatory Feeding for Cellulose‐Mediated Dietary Dilution in a Generalist Caterpillar.” Physiological Entomology 29: 108–117.

[ecy70383-bib-0040] Lemoine, N. P. , S. T. Giery , and D. E. Burkepile . 2014. “Differing Nutritional Constraints of Consumers across Ecosystems.” Oecologia 174: 1367–1376. 24380968 10.1007/s00442-013-2860-z

[ecy70383-bib-0041] Leroux, S. J. , E. V. Wal , Y. F. Wiersma , L. Charron , J. D. Ebel , N. M. Ellis , C. Hart , et al. 2017. “Stoichiometric Distribution Models: Ecological Stoichiometry at the Landscape Extent.” Ecology Letters 20: 1495–1506. 29027338 10.1111/ele.12859

[ecy70383-bib-0042] Levey, D. J. , and W. H. Karasov . 1989. “Digestive Responses of Temperate Birds Switched to Fruit or Insect Diets.” The Auk 106: 675–686.

[ecy70383-bib-0043] Lindroth, R. L. , K. A. Klein , J. D. C. Hemming , and A. M. Feuker . 1997. “Variation in Temperature and Dietary Nitrogen Affect Performance of the Gypsy Moth (*Lymantria dispar* L.).” Physiological Entomology 22: 55–64.

[ecy70383-bib-0044] Lu, J. , X. Zhao , S. Wang , S. Feng , Z. Ning , R. Wang , X. Chen , H. Zhao , and M. Chen . 2023. “Untangling the Influence of Abiotic and Biotic Factors on Leaf C, N, and P Stoichiometry along a Desert‐Grassland Transition Zone in Northern China.” Science of the Total Environment 884: 163902. 37137371 10.1016/j.scitotenv.2023.163902

[ecy70383-bib-0045] Mattson, W. J. 1980. “Herbivory in Relation to Plant Nitrogen Content.” Annual Review of Ecology and Systematics 11: 119–161.

[ecy70383-bib-0046] McArt, S. H. , D. E. Spalinger , W. B. Collins , E. R. Schoen , T. Stevenson , and M. Bucho . 2009. “Summer Dietary Nitrogen Availability as a Potential Bottom‐Up Constraint on Moose in South‐Central Alaska.” Ecology 90: 1400–1411. 19537559 10.1890/08-1435.1

[ecy70383-bib-0047] McLeod, A. M. , S. Leroux , C. L. Little , F. Massol , E. Vander Wal , Y. F. Wiersma , I. Gounand , N. Loeuille , and E. Harvey . 2025. “Quantifying Elemental Diversity to Study Landscape Ecosystem Function.” Trends in Ecology & Evolution 40: 57–66. 39419673 10.1016/j.tree.2024.09.007

[ecy70383-bib-0048] Moorman, C. E. , L. T. Bowen , J. C. Kilgo , C. E. Sorenson , J. L. Hanula , S. Horn , and M. D. Ulyshen . 2007. “Seasonal Diets of Insectivorous Birds Using Canopy Gaps in a Bottomland Forest.” Journal of Field Ornithology 78: 11–20.

[ecy70383-bib-0049] Murakami, M. , T. Ichie , and T. Hirao . 2008. “Beta‐Diversity of Lepidopteran Larval Communities in a Japanese Temperate Forest: Effects of Phenology and Tree Species.” Ecological Research 23: 179–187.

[ecy70383-bib-0050] Ontario Ministry of Natural Resources . 2023. “Ontario Land Cover.” https://geohub.lio.gov.on.ca/documents/667367a759214a089917adccdbae7cb2/about.

[ecy70383-bib-0051] Pedersen, E. J. , D. L. Miller , G. L. Simpson , and N. Ross . 2019. “Hierarchical Generalized Additive Models in Ecology: An Introduction with mgcv.” PeerJ 7: e6876. 31179172 10.7717/peerj.6876 PMC6542350

[ecy70383-bib-0052] Persson, J. , P. Fink , A. Goto , J. M. Hood , J. Jonas , and S. Kato . 2010. “To be or Not to be What you Eat: Regulation of Stoichiometric Homeostasis Among Autotrophs and Heterotrophs.” Oikos 119: 741–751.

[ecy70383-bib-0053] Prather, R. M. , K. Castillioni , M. Kaspari , L. Souza , C. M. Prather , R. W. Reihart , and E. A. R. Welti . 2020. “Micronutrients Enhance Macronutrient Effects in a Meta‐Analysis of Grassland Arthropod Abundance.” Global Ecology and Biogeography 29: 2273–2288.

[ecy70383-bib-0054] R Development Core Team . 2024. R: A Language and Environment for Statistical Computing. Vienna, Austria: R Foundation for Statistical Computing. https://www.r-project.org.

[ecy70383-bib-0055] Raubenheimer, D. , K. P. Lee , and S. J. Simpson . 2005. “Does Bertrand's Rule Apply to Macronutrients?” Proceedings of the Royal Society B 272: 2429–2434. 16243690 10.1098/rspb.2005.3271 PMC1559972

[ecy70383-bib-0056] Raubenheimer, D. , and S. J. Simpson . 2003. “Nutrient Balancing in Grasshoppers: Behavioural and Physiological Correlates of Dietary Breadth.” Journal of Experimental Biology 206: 1669–1681. 12682099 10.1242/jeb.00336

[ecy70383-bib-0057] Raubenheimer, D. , S. J. Simpson , and D. Mayntz . 2009. “Nutrition, Ecology and Nutritional Ecology: Toward an Integrated Framework.” Functional Ecology 23: 4–16.

[ecy70383-bib-0058] Razeng, E. , and D. M. Watson . 2015. “Nutritional Composition of the Preferred Prey of Insectivorous Birds: Popularity Reflects Quality.” Journal of Avian Biology 46: 89–96.

[ecy70383-bib-0059] Reynolds, S. J. , S. J. Schoech , and R. Bowman . 2003. “Diet Quality During Pre‐Laying and Nestling Periods Influences Growth and Survival of Florida Scrub‐Jay (*Aphelocoma coerulescens*) Chicks.” Journal of Zoology 261: 217–226.

[ecy70383-bib-0060] Ritchie, M. E. 2000. “Nitrogen Limitation and Trophic vs. Abiotic Influences on Insect Herbivores in a Temperate Grassland.” Ecology 81: 1601–1612.

[ecy70383-bib-0061] Rizzuto, M. , S. J. Leroux , E. Vander Wal , I. C. Richmond , T. R. Heckford , J. Balluffi‐Fry , and Y. F. Wiersma . 2021. “Forage Stoichiometry Predicts the Home Range Size of a Small Terrestrial Herbivore.” Oecologia 197: 327–338. 34131817 10.1007/s00442-021-04965-0

[ecy70383-bib-0062] Rosenblatt, A. E. 2018. “Shifts in Plant Nutrient Content in Combined Warming and Drought Scenarios May Alter Reproductive Fitness across Trophic Levels.” Oikos 127: 1853–1862.

[ecy70383-bib-0063] Rosenblatt, A. E. , and O. J. Schmitz . 2016. “Climate Change, Nutrition, and Bottom‐Up and Top‐Down Food Web Processes.” Trends in Ecology & Evolution 31: 965–975. 27726943 10.1016/j.tree.2016.09.009

[ecy70383-bib-0064] Rytkönen, S. , E. J. Vesterinen , C. Westerduin , T. Leviäkangas , E. Vatka , M. Mutanen , P. Välimäki , M. Hukkanen , M. Suokas , and M. Orell . 2019. “From Feces to Data: A Metabarcoding Method for Analyzing Consumed and Available Prey in a Bird‐Insect Food Web.” Ecology and Evolution 9: 631–639. 30680143 10.1002/ece3.4787 PMC6342092

[ecy70383-bib-0065] Sanders‐DeMott, R. , R. McNellis , M. Jabouri , and P. H. Templer . 2018. “Snow Depth, Soil Temperature and Plant–Herbivore Interactions Mediate Plant Response to Climate Change.” Journal of Ecology 106: 1508–1519.

[ecy70383-bib-0066] Sardans, J. , I. A. Janssens , P. Ciais , M. Obersteiner , and J. Peñuelas . 2021. “Recent Advances and Future Research in Ecological Stoichiometry.” Perspectives in Plant Ecology, Evolution and Systematics 50: 125611.

[ecy70383-bib-0067] Sardans, J. , A. Rivas‐Ubach , and J. Peñuelas . 2011. “Factors Affecting Nutrient Concentration and Stoichiometry of Forest Trees in Catalonia (NE Spain).” Forest Ecology and Management 262: 2024–2034.

[ecy70383-bib-0068] Scriber, J. M. , and F. Slansky . 1981. “The Nutritional Ecology of Immature Insects.” Annual Review of Entomology 26: 183–211.

[ecy70383-bib-0069] Seemann, J. R. , T. D. Sharkey , J. Wang , and C. B. Osmond . 1987. “Environmental Effects on Photosynthesis, Nitrogen‐Use Efficiency, and Metabolite Pools in Leaves of Sun and Shade Plants.” Plant Physiology 84: 796–802. 16665524 10.1104/pp.84.3.796 PMC1056672

[ecy70383-bib-0070] Shipley, J. R. , C. W. Twining , M. Mathieu‐Resuge , T. P. Parmar , M. Kainz , D. Martin‐Creuzburg , C. Weber , D. W. Winkler , C. H. Graham , and B. Matthews . 2022. “Climate Change Shifts the Timing of Nutritional Flux from Aquatic Insects.” Current Biology 32: 1342–1349.e3. 35172126 10.1016/j.cub.2022.01.057

[ecy70383-bib-0071] Sierro, A. , R. Arlettaz , B. Naef‐Daenzer , S. Strebel , and N. Zbinden . 2001. “Habitat Use and Foraging Ecology of the Nightjar (*Caprimulgus europaeus*) in the Swiss Alps: Towards a Conservation Scheme.” Biological Conservation 98: 325–331.

[ecy70383-bib-0072] Simpson, S. J. , R. M. Sibly , K. P. Lee , S. T. Behmer , and D. Raubenheimer . 2004. “Optimal Foraging When Regulating Intake of Multiple Nutrients.” Animal Behaviour 68: 1299–1311.

[ecy70383-bib-0073] Sitters, J. , E. S. Bakker , M. P. Veldhuis , G. F. Veen , H. Olde Venterink , and M. J. Vanni . 2017. “The Stoichiometry of Nutrient Release by Terrestrial Herbivores and Its Ecosystem Consequences.” Frontiers in Earth Science 5: 32.

[ecy70383-bib-0074] Śmielak, M. 2024. “moonlit: Predicting Moonlight Intensity for Given Time and Location.” R package Version 0.1.0. https://github.com/msmielak/moonlit.

[ecy70383-bib-0075] Śmielak, M. K. 2023. “Biologically Meaningful Moonlight Measures and Their Application in Ecological Research.” Behavioral Ecology and Sociobiology 77: 21.

[ecy70383-bib-0076] Souza‐Cole, I. , M. P. Ward , R. L. Mau , J. T. Foster , and T. J. Benson . 2022. “Eastern Whip‐Poor‐Will Abundance Declines with Urban Land Cover and Increases with Moth Abundance in the American Midwest.” Ornithological Applications 124: duac032.

[ecy70383-bib-0077] Stan Development Team . 2024. “Stan Modeling Language Users Guide and Reference Manual.” https://mc-stan.org.

[ecy70383-bib-0078] Sterner, R. W. , and J. J. Elser . 2002. Ecological Stoichiometry: The Biology of Elements from Molecules to the Biosphere. Princeton, NJ: Princeton University Press.

[ecy70383-bib-0079] Studier, E. H. , J. O. Keeler , and S. H. Sevick . 1991. “Nutrient Composition of Caterpillars, Pupae, Cocoons and Adults of the Eastern Tent Moth, *Malacosoma americanum* (Lepidoptera: Lasiocampidae).” Comparative Biochemistry and Physiology Part A: Physiology 100: 1041–1043.

[ecy70383-bib-0080] Sun, J. , B. Liu , Y. You , W. Li , M. Liu , H. Shang , and J.‐S. He . 2019. “Solar Radiation Regulates the Leaf Nitrogen and Phosphorus Stoichiometry Across Alpine Meadows of the Tibetan Plateau.” Agricultural and Forest Meteorology 271: 92–101.

[ecy70383-bib-0081] Suzuki‐Ohno, Y. , M. Kawata , and J. Urabe . 2012. “Optimal Feeding under Stoichiometric Constraints: A Model of Compensatory Feeding with Functional Response.” Oikos 121: 569–578.

[ecy70383-bib-0082] Tsahar, E. , Z. Arad , I. Izhaki , and C. M. del Rio . 2006. “Do Nectar‐ and Fruit‐Eating Birds Have Lower Nitrogen Requirements than Omnivores? An Allometric Test.” The Auk 123: 1004–1012.

[ecy70383-bib-0083] Twining, C. W. , J. R. Shipley , and B. Matthews . 2022. “Climate Change Creates Nutritional Phenological Mismatches.” Trends in Ecology & Evolution 37: 736–739. 35811171 10.1016/j.tree.2022.06.009

[ecy70383-bib-0084] Wäckers, F. L. , J. Romeis , and P. Van Rijn . 2007. “Nectar and Pollen Feeding by Insect Herbivores and Implications for Multitrophic Interactions.” Annual Review of Entomology 52: 301–323. 10.1146/annurev.ento.52.110405.091352 16972766

[ecy70383-bib-0085] Walsh, N. E. , T. R. McCabe , J. M. Welker , and A. N. Parsons . 1997. “Experimental Manipulations of Snow‐Depth: Effects on Nutrient Content of Caribou Forage.” Global Change Biology 3: 158–164.

[ecy70383-bib-0086] Wheelwright, N. T. 1986. “The Diet of American Robins: An Analysis of U.S. Biological Survey Records.” The Auk 103: 710–725.

[ecy70383-bib-0087] White, T. C. R. 1984. “The Abundance of Invertebrate Herbivores in Relation to the Availability of Nitrogen in Stressed Food Plants.” Oecologia 63: 90–105. 28311171 10.1007/BF00379790

[ecy70383-bib-0088] Xie, H. , M. Yu , and X. Cheng . 2018. “Leaf Non‐structural Carbohydrate Allocation and C:N:P Stoichiometry in Response to Light Acclimation in Seedlings of Two Subtropical Shade‐Tolerant Tree Species.” Plant Physiology and Biochemistry 124: 146–154. 29366973 10.1016/j.plaphy.2018.01.013

[ecy70383-bib-0089] Zhang, B. , H. Chen , M. Deng , X. Li , T. Chen , L. Liu , S. Scheu , and S. Wang . 2022. “Multidimensional Stoichiometric Mismatch Explains Differences in Detritivore Biomass Across Three Forest Types.” Journal of Animal Ecology 92: 454–465. 36477808 10.1111/1365-2656.13859

[ecy70383-bib-0090] Zhu, C. , M. Zhang , Y. Chen , D. Yin , D. He , S. Fang , M. D. F. Ellwood , and C. Chu . 2021. “Plant‐Caterpillar Food Web: Integrating Leaf Stoichiometry and Phylogeny.” Ecological Entomology 46: 1026–1035.

