# Supplementary material for: Seasonal stoichiometry of terrestrial consumer–resource interactions
Source: Ecology. 2026 Apr 9;107(4):e70383. doi: 10.1002/ecy.70383 (PMC13063215; doi:10.1002/ecy.70383)
Supplement: Supplementary file 1 — Appendix S1. [file ECY-107-e70383-s002.pdf]

**Appendix S1**

**Seasonal stoichiometry of terrestrial consumer–resource interactions**

Richard E. Feldman, Anna Singh, Paul C. Frost

*Ecology*

Note: we used Microsoft Copilot to help write the correct statistical notation. However, coding and running the models were all done by the authors.

Section S1. Modeling the change in C, N, and C:N over the season for alvar leaves, forest edge leaves, and moths.

Section S1.1. For the proportion of C or N in sample type $i$ (where $i$ = alvar leaf, edge leaf, moth) taken during week $j$ at sampling location $k$

$$\begin{aligned}
\mathrm{y}_{ijk} &\sim \mathrm{Beta}(\mu_{ijk}, \phi_{ijk}) \\
\mu_{ijk} &= \beta_{0k} Type_{ik} + \beta_{1k} Type_{ik} Week_j \\
\begin{bmatrix} \beta_{0k} \\ \beta_{1k} \end{bmatrix} &\sim \mathrm{MVNormal}\left( \left( \begin{bmatrix} \beta_0 \\ \beta_1 \end{bmatrix}, \mathbf{\Sigma} \right) \right) \\
\mathbf{\Sigma} &= \begin{bmatrix} \sigma_0 & 0 \\ 0 & \sigma_1 \end{bmatrix} \mathbf{R} \begin{bmatrix} \sigma_0 & 0 \\ 0 & \sigma_1 \end{bmatrix} \\
\mathbf{R} &= \begin{bmatrix} 1 & \rho_{12} \\ \rho_{21} & 1 \end{bmatrix} \\
\beta_0 &\sim \mathrm{Normal}(0, 1) \\
\beta_1 &\sim \mathrm{Normal}(0, 1) \\
\phi &\sim \mathrm{Gamma}(4, 0.1) \\
\sigma_0 &\sim \mathrm{Exponential}(1) \\
\sigma_1 &\sim \mathrm{Exponential}(1) \\
\mathbf{R} &\sim \mathrm{LKJcorr}(2)
\end{aligned}$$

Section S1.2. For C:N in sample type $i$ (where $i$ = alvar leaf, edge leaf, moth) taken during week $j$ at sampling location $k$

$$\begin{aligned}
\mathrm{y}_{ijk} &\sim \mathrm{Gamma}(\mu_{ijk}, \lambda_{ijk}) \\
\log(\mu_{ijk}) &= \beta_{0k} Type_{ik} + \beta_{1k} Type_{ik} Week_j \\
\begin{bmatrix} \beta_{0k} \\ \beta_{1k} \end{bmatrix} &\sim \mathrm{MVNormal}\left( \left( \begin{bmatrix} \beta_0 \\ \beta_1 \end{bmatrix}, \mathbf{\Sigma} \right) \right) \\
\mathbf{\Sigma} &= \begin{bmatrix} \sigma_0 & 0 \\ 0 & \sigma_1 \end{bmatrix} \mathbf{R} \begin{bmatrix} \sigma_0 & 0 \\ 0 & \sigma_1 \end{bmatrix} \\
\mathbf{R} &= \begin{bmatrix} 1 & \rho_{12} \\ \rho_{21} & 1 \end{bmatrix} \\
\beta_0 &\sim \mathrm{Normal}(-0.223, 0.668) \\
\beta_1 &\sim \mathrm{Normal}(0, 0.25) \\
\lambda &\sim \mathrm{Gamma}(0.01, 0.01) \\
\sigma_0 &\sim \mathrm{Exponential}(1) \\
\sigma_1 &\sim \mathrm{Exponential}(1) \\
\mathbf{R} &\sim \mathrm{LKJcorr}(2)
\end{aligned}$$

Section S2. Modeling the change in C, N, and C:N across sampling locations (combination of sample point and habitat (alvar or edge)) with different amounts of light.

Section S2.1. For the proportion of C or N in leaf $i$ taken during week $j$ at sampling location $k$

$$
\begin{aligned}
\mathrm{y}_{ijk} &\sim \mathrm{Beta}(\mu_{ijk}, \phi_{ijk}) \\
\mu_{ijk} &= \alpha + \beta_1 Light_{ik} + \omega_j + \upsilon_k \\
\omega_j &\sim \mathrm{Normal}(0, \sigma_j) \\
\upsilon_k &\sim \mathrm{Normal}(0, \sigma_k) \\
\alpha &\sim \mathrm{Normal}(0, 1) \\
\beta_1 &\sim \mathrm{Normal}(0, 1) \\
\phi &\sim \mathrm{Gamma}(4, 0.1) \\
\sigma_j &\sim \mathrm{Exponential}(1) \\
\sigma_k &\sim \mathrm{Exponential}(1)
\end{aligned}
$$

Section S2.2. For C:N of leaf $i$ taken during week $j$ at sampling location $k$

$$
\begin{aligned}
\mathrm{y}_{ijk} &\sim \mathrm{Normal}(\mu_{ijk}, \sigma) \\
\mu_{ij} &= \alpha + \beta_1 Light_{ik} + \omega_j + \upsilon_k \\
\omega_j &\sim \mathrm{Normal}(0, \sigma_j) \\
\upsilon_k &\sim \mathrm{Normal}(0, \sigma_k) \\
\alpha &\sim \mathrm{Normal}(0, 1) \\
\beta_1 &\sim \mathrm{Normal}(0, 0.25) \\
\sigma_j &\sim \mathrm{Exponential}(1) \\
\sigma_k &\sim \mathrm{Exponential}(1)
\end{aligned}
$$

Section S3. Modeling the change in per-individual moth body mass and total moth abundance over the season and in relation to foliar C:N while adjusting for nightly temperature, windspeed, and moonlight intensity. The temporal relationship is a smoothed spline with a basis dimension k = 5 and a factor-smooth interaction between time and the specific moth trap. We also include trap as a random intercept

Section S3.1. For per-individual moth body mass measured in week $i$ and trap $j$ with spline basis functions $\mathrm{B_m}$

$$
\begin{aligned}
y_{ij} &\sim \text{Lognormal}(\mu_{ij}, \sigma) \\
\mu_{ij} &= \alpha_j + \sum_{m=1}^{5} \beta_{1m}\, B_{1m} Week_i \\
&\quad + \beta_2 \text{Ratio}_{ij} + \beta_3 \text{Temp}_{ij} + \beta_4 \text{Wind}_{ij} + \beta_5 \text{Moonlight}_{ij} \\
&\quad + \sum_{n=1}^{N} \sum_{m=1}^{5} \beta_{5mn}\, B_{5mn} \text{Week}_i \text{Trap}_j \\
\alpha_j &\sim \text{Normal}(\alpha, \epsilon) \\
\beta &\sim \text{Normal}(0, 1) \\
\beta_m &\sim \text{Normal}(0, sds) \\
\sigma &\sim \text{Normal}(log(0.832), 0.5) \\
\alpha &\sim \text{Normal}(log(100), 0.5) \\
\epsilon &\sim \text{Exponential}(2) \\
\text{sds} &\sim \text{Exponential}(1)
\end{aligned}
$$

Section S3.2. For total moth abundance measured in week $i$ and trap $j$ with spline basis functions $\mathrm{B_m}$ and right censored at 50 individuals

For observation $y_{ij}$:

$$
y_{ij}^{obs} = \begin{cases} y_{ij}, & \text{if } y_{ij} < 50 \\ 50, & \text{if } y_{ij} \geq 50 \end{cases}
$$

The likelihood becomes:

$$
p(y_{ij} \mid \mu_{ij}, \sigma) = \begin{cases} \text{Normal}(y_{ij} \mid \mu_{ij}, \sigma), & y_{ij} < 50, \\ 1 - F(50 \mid \mu_{ij}, \sigma), & y_{ij} \geq 50, \end{cases}
$$

Where $F(\cdot)$ is the cumulative distribution function of the normal distribution.

$$
\begin{aligned}
\mu_{ij} &= \alpha_j + \sum_{m=1}^{5} \beta_{1m}\, B_{1m} Week_i \\
&\quad + \beta_2 \text{Ratio}_{ij} + \beta_3 \text{Temp}_{ij} + \beta_4 \text{Wind}_{ij} + \beta_5 \text{Moonlight}_{ij} \\
&\quad + \sum_{n=1}^{N} \sum_{m=1}^{5} \beta_{5mn}\, B_{5mn} \text{Week}_i \text{Trap}_j \\
\alpha_j &\sim \text{Normal}(\alpha, \epsilon) \\
\beta &\sim \text{Normal}(0, 1) \\
\beta_m &\sim \text{Normal}(0, sds) \\
\sigma &\sim \text{StudentT}(\nu = 3, \mu = 0, \tau = 50.7) \\
\alpha &\sim \text{Normal}(36, 10) \\
\epsilon &\sim \text{Exponential}(2) \\
\text{sds} &\sim \text{Exponential}(1)
\end{aligned}
$$
