# Supplementary material for: Seasonal stoichiometry of terrestrial consumer–resource interactions
Source: Ecology. 2026 Apr 9;107(4):e70383. doi: 10.1002/ecy.70383 (PMC13063215; doi:10.1002/ecy.70383)
Supplement: Supplementary file 2 — Appendix S2. [file ECY-107-e70383-s001.pdf]

Appendix S2

Seasonal stoichiometry of terrestrial consumer–resource interactions

Richard E. Feldman, Anna Singh, Paul C. Frost

Ecology

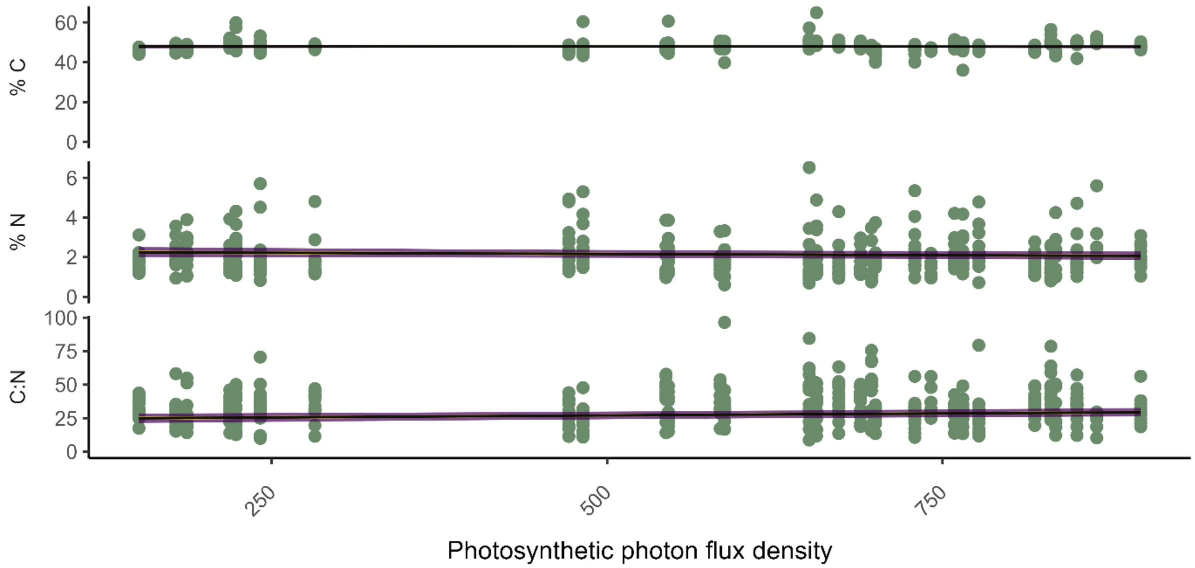

Figure S1. Change along a light gradient in the carbon and nitrogen content and C:N ratios for leaves taken from plants growing in the open alvar and forest edge. The yellow and purple shadings represent the 50% and 95% credible intervals of the posterior predictive distribution. The black lines are the medians of the posterior predictive distributions. The green points are the observed contents and ratios for the leaf from each of 30 sampling points (15 points for each of forest and edge leaves) at different time points across the season. Each sampling point only has one light level to give the general condition, and not week-to-week fluctuations based on time of day and cloud cover.

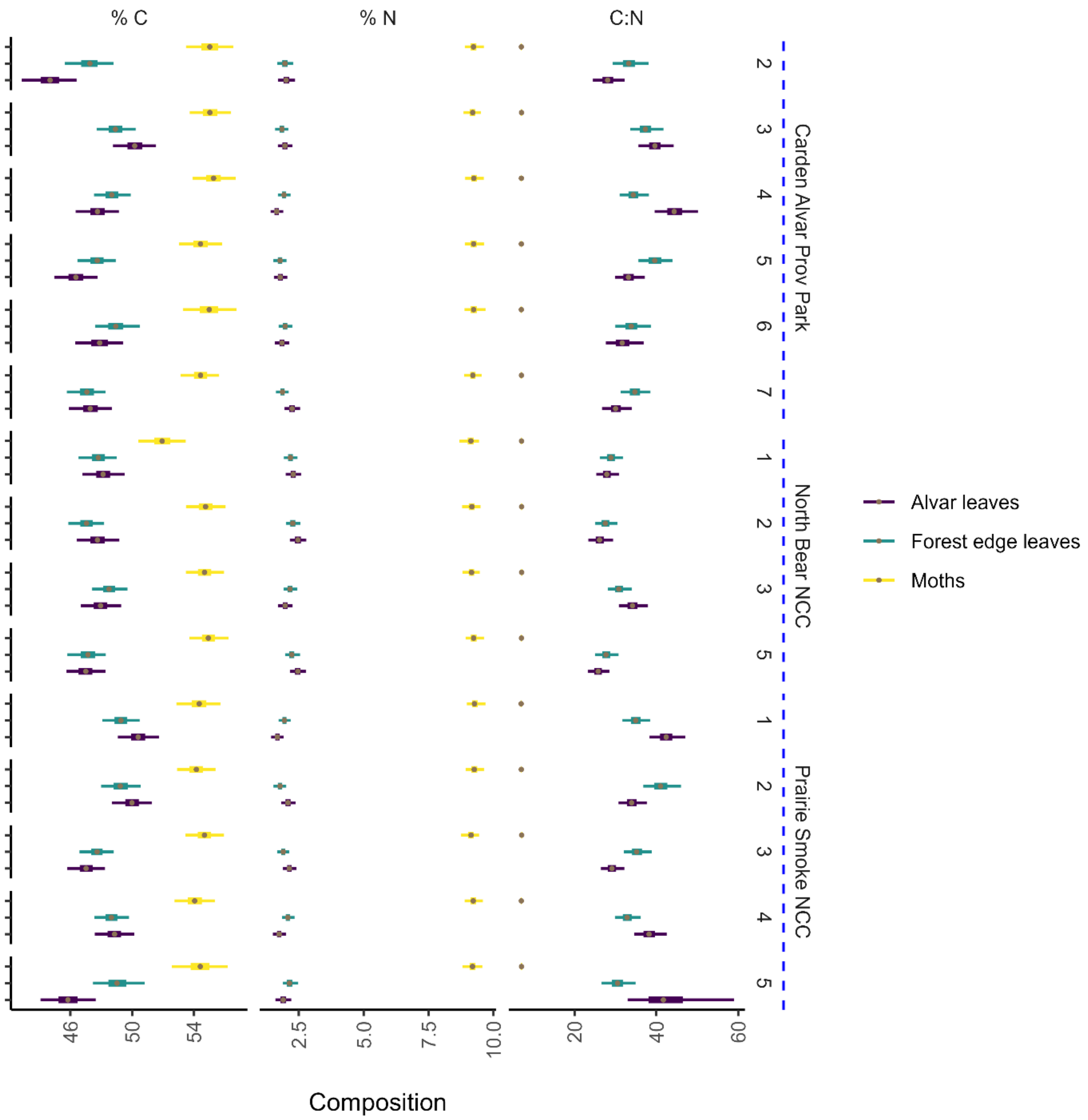

Figure S2. Variability across sampling points in carbon and nitrogen composition and C:N ratios for leaves taken from plants growing in the open alvar and forest edge and from moths. The thick and thin bars represent 50% and 95% credible intervals of the posterior distribution of the estimates averaged across all sampling weeks. The points are the medians of the posterior distributions.

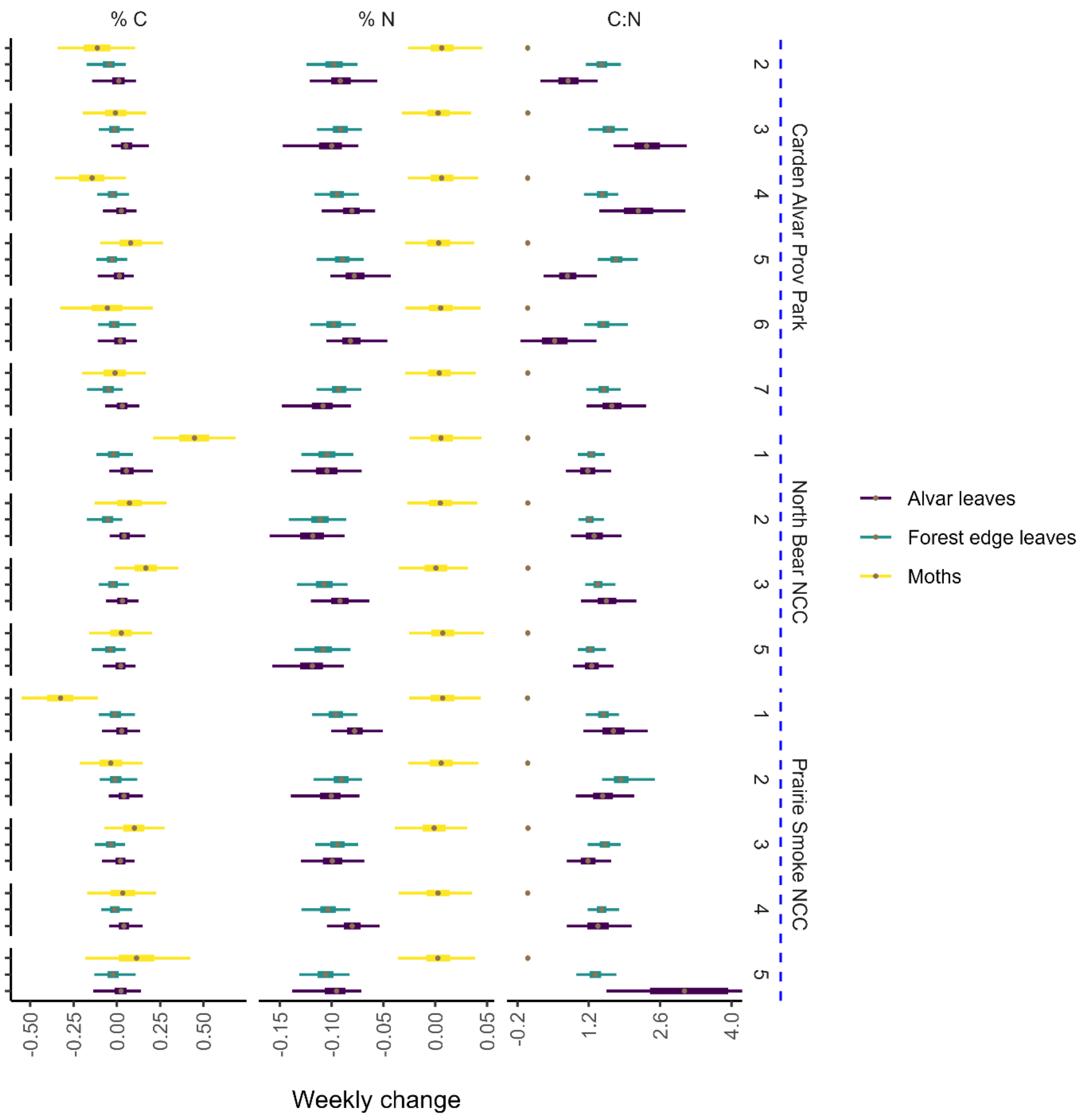

Figure S3. Variability across sampling points in the seasonal change in carbon and nitrogen composition and C:N ratios for leaves taken from plants growing in the open alvar and forest edge and from moths. The thick and thin bars represent 50% and 95% credible intervals of the posterior distribution of the slope estimate of weekly change. The points are the medians of the posterior distributions.

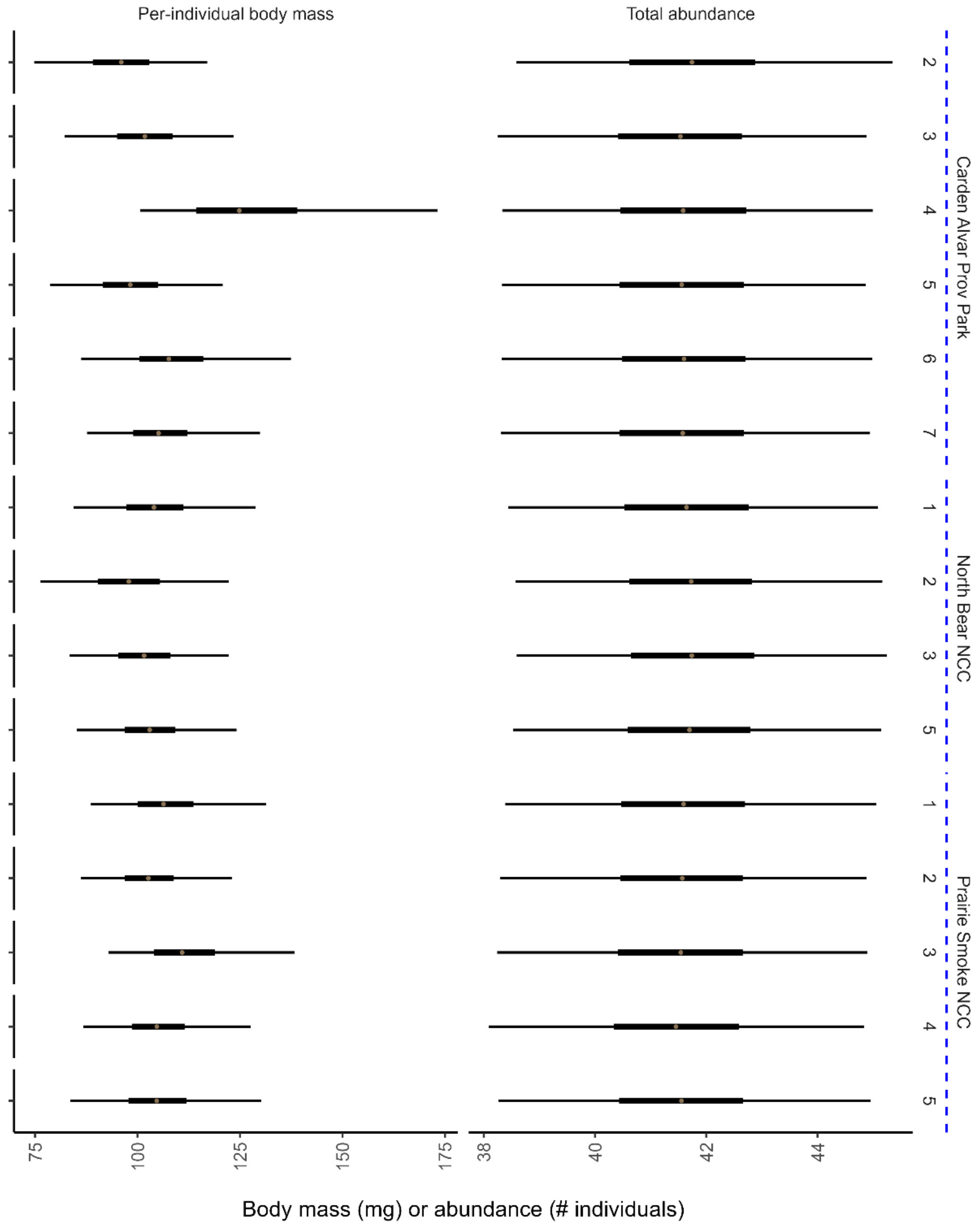

Figure S4. Variability across sampling points in per-individual moth biomass and total moth abundance. The thick and thin bars represent 50% and 95% credible intervals of the posterior distribution of the estimates averaged across the gradient of foliar C:N ratios. The points are the medians of the posterior distributions.
