# Supplementary material for: Seasonal stoichiometry of terrestrial consumer–resource interactions
Source: Ecology. 2026 Apr 9;107(4):e70383. doi: 10.1002/ecy.70383 (PMC13063215; doi:10.1002/ecy.70383)
Supplement: Supplementary file 3 — Appendix S3. [file ECY-107-e70383-s003.pdf]

Appendix S3

Seasonal stoichiometry of terrestrial consumer–resource interactions

Richard E. Feldman, Anna Singh, Paul C. Frost

Ecology

Load packages

```
library(pacman)

p_load(
  brms, tidyverse, tidybayes, viridis, bayesplot, scales,
  marginaleffects, ggh4x, patchwork, ggpubr, performance,
  broom.mixed, ggdist, vegan, otuSummary, cmdstanr, styler,
  weathercan, moonlit, suntools, kableExtra, glue, lubridate, tinytext
)

rhat <- brms::rhat
register_knitr_engine(override = TRUE)
knitr::opts_chunk$set(tidy = "styler")
```

Import data

```
ratios_1 <- read_csv("data/ewpw_ratios_complete.csv") %>%
  rename(
    Trap = "Trap Location", Type = "Leaf Type", Date = "Sample Date",
    N = "%N", C = "%C", Ratio = "C/N  ratio (by mol)"
  ) %>%
  select(-c(10:12))

ratios_2 <- ratios_1 %>%
  filter(
    !Well %in% c("NO MOTHS"),
    !Date %in% c("unknown", "Incorrect date")
  ) %>%
  mutate(Week = as.numeric(Week)) %>%
  mutate(
    Date = as.Date(paste(Date, "2023"), format = "%d-%b %Y"),
    Site = str_sub(Trap, 1, 2),
    Week_2 = rescale(Week)
  ) %>%
  filter(Trap != "NB4") %>%
  mutate(N = N / 100, C = C / 100)

ratios_3 <- ratios_2 %>%
  pivot_longer(c("N", "C", "Ratio"),
    names_to = "Component",
```

```
    values_to = "Ratio"
  )
```

Set-up analyses

```
all_analyses <- ratios_3 %>%
  group_by(Component) %>%
  nest() %>%
  mutate(
    Model =
      list(bf(Ratio ~ 0 + Type + s(Week_2, by = Type, k = 5) +
        s(Week_2, Trap, k = 5, bs = "fs"))),
    Family = case_when(
      Component %in% c("N", "C") ~ list(Beta()),
      .default = list(Gamma(link = "log"))
    ),
    Priors = case_when(
      Component == "Ratio" ~ list(c(
        prior(normal(-0.223, 0.668), class = b),
        prior(exponential(1), class = sds)
      )),
      .default = list(c(
        prior(normal(0, 1), class = b),
        prior(gamma(4, 0.1), class = phi),
        prior(exponential(1), class = sds)
      ))
    )
  )
```

Run prior predictive checks

```
all_priors <- all_analyses %>%
  mutate(Prior_sampling = pmap(
    list(
      Model, data, Family, Priors, Component
    ),
    function(first, second, third, fourth, fifth) {
      brm(
        formula = first,
        data = second,
        family = third,
        prior = fourth,
        sample_prior = "only",
        iter = 2000,
        warmup = 1000,
        chains = 4,
        cores = 4,
        control = list(
          adapt_delta = 0.99,
          max_treedepth = 15
        ),
```

```
        file = paste("PriorGAM", fifth,
          sep = "_"
        ),
        backend = "cmdstanr"
      )
    }
  ))
```

Produce the figure of prior predictive checks

```
all_priors <- readRDS("all_priors_GAM.rds") %>%
  mutate(prior_checks = map(
    Prior_sampling,
    function(first) {
      pp_check(first,
        type = "stat_grouped",
        group = "Type", prefix = "ppd"
      )
    }
  ))

wrap_plots(all_priors$prior_checks,
  ncol = 1, nrow = 3,
  axis_titles = "collect_x"
)
```

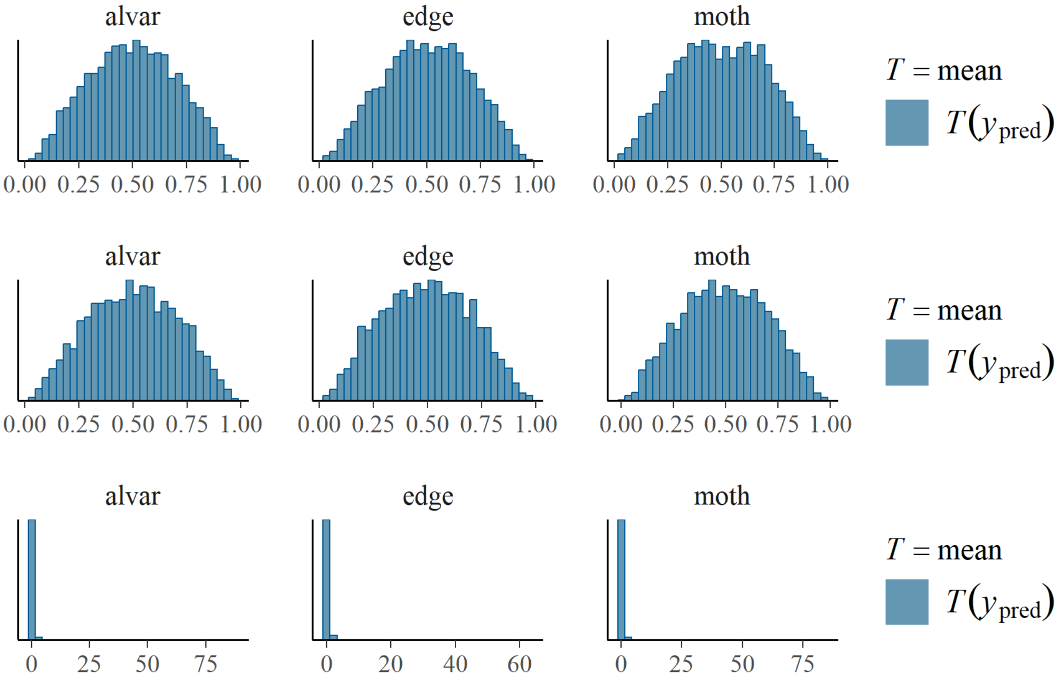

Prior predictive checks for generalized additive models for seasonal patterns of C (top), N (middle), and C:N (bottom) for each of alvar leaves, edge leaves, and moths.

Return posteriors

```
all_posteriors <- all_analyses %>%
  mutate(Posterior_sampling = pmap(
    list(
      Model,
      data,
      Family,
      Priors,
      Component
    ),
    function(first,
             second,
             third,
             fourth,
             fifth) {
      brm(
        formula = first,
        data = second,
        family = third,
        prior = fourth,
        iter = 8000,
        warmup = 4000,
        chains = 4,
```

```
        cores = 4,
        control = list(
          adapt_delta = 0.99,
          max_treedepth = 15
        ),
        file = paste("PosteriorGAM", fifth,
          sep = "_"
        ),
        backend = "cmdstanr"
      )
    }
  ))
```

Run posterior predictive checks

```
all_posteriors <- readRDS("all_posteriors_GAM.rds") %>%
  mutate(posteriors_checks = map(
    Posterior_sampling,
    function(first) {
      pp_check(first,
        type = "stat_grouped",
        group = "Type", prefix = "ppc"
      )
    }
  ))

wrap_plots(all_posteriors$posteriors_checks,
  ncol = 1, nrow = 3,
  axis_titles = "collect_x"
)
```

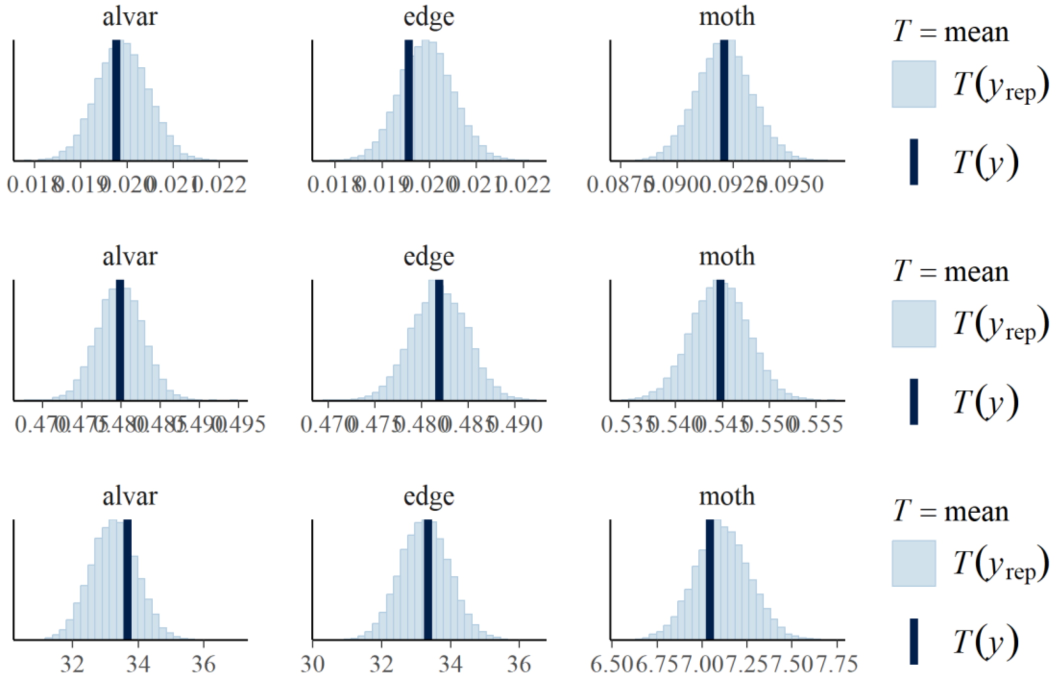

Posterior predictive checks for generalized additive models for seasonal patterns of C (top), N (middle), and C:N (bottom) for each of alvar leaves, edge leaves, and moths.

Function to predict elemental composition and ratios across the growing season

```
predict_data <- ratios_3 %>%
  group_by(Week, Week_2) %>%
  slice_min(Date) %>%
  select(Week, Week_2, Date) %>%
  distinct() %>%
  full_join(tibble(Week = seq(min(ratios_3$Week), max(ratios_3$Week), 1)))
%>%
  ungroup() %>%
  mutate(Week_2 = rescale(Week)) %>%
  arrange(Week_2) %>%
  mutate(Date = if_else(is.na(Date), lag(Date, n = 1) + 7, Date)) %>%
  mutate(Date = if_else(is.na(Date), lag(Date, n = 2) + 14, Date)) %>%
  expand(nesting(Week, Week_2, Date),
    Trap = unique(ratios_3$Trap),
    Type = unique(ratios_3$Type)
  )

get_predictions <- function(x) {
  preds <- avg_predictions(x,
    newdata = predict_data,
    by = c("Week_2", "Type", "Date")
  ) %>%
```

```
    posterior_draws()
}
```

Run prediction function

```
all_posteriors <- all_posteriors %>%
  mutate(
    predictions = map(Posterior_sampling, get_predictions)
  ) %>%
  ungroup()
```

Return predicted elemental composition and ratios at beginning, middle, and end of season

```
all_posteriors %>%
  filter(Component == "Ratio") %>%
  unnest(predictions) %>%
  filter(Type == "moth") %>%
  group_by(Week_2) %>%
  median_qi(draw) %>%
  slice(c(1, 12, 23))

# A tibble: 3 × 7
  Week_2  draw .lower .upper .width .point .interval
   <dbl> <dbl>  <dbl>  <dbl>  <dbl> <chr>  <chr>
1    0    6.32   5.73   6.96   0.95 median qi
2    0.5  7.43   7.08   7.81   0.95 median qi
3    1    6.90   6.37   7.49   0.95 median qi
```

Figure of seasonal trends in elemental composition and ratios

```
phenology_plotting_stoich <- function(the_component, the_type,
the_predictions) {
  if (the_component == "Ratio") {
    ylabel <- "C:N\n"
  }
  if (the_component == "C") {
    ylabel <- "% C\n"
  }
  if (the_component == "N") {
    ylabel <- "% N\n"
  }

  phenology_plot <- the_predictions %>%
    group_by(Date) %>%
    median_qi(draw, .width = c(0.5, 0.95)) %>%
    ggplot(aes(x = Date, y = draw, ymin = .lower, ymax = .upper)) +
    geom_point(
      data = ratios_3 %>%
        filter(
          Component == the_component,
```

```
          Type == the_type
        ) %>%
        rename(draw = Ratio) %>%
        mutate(draw = ifelse(Component == "Ratio", draw, draw * 100)) %>%
        mutate(.lower = 0, .upper = 0),
      aes(x = Date, y = draw),
      colour = "darkseagreen4", size = 2
    ) +
    geom_lineribbon(alpha = 0.7, linewidth = 0.5) +
    # scale_y_continuous(name = ylabel) +
    scale_x_date("", date_breaks = "2 weeks", date_labels = "%b-%d") +
    scale_fill_viridis(discrete = TRUE) +
    theme_classic()

  if (the_type == "moth") {
    phenology_plot <- phenology_plot +
      theme(
        axis.text.x = element_text(angle = 90, vjust = 0.5, hjust = 1),
        legend.position = "none"
      )
  } else {
    phenology_plot <- phenology_plot +
      theme(
        axis.text.x = element_blank(),
        legend.position = "none"
      )
  }

  if (the_type == "edge") {
    phenology_plot <- phenology_plot +
      scale_y_continuous(name = paste0(ylabel, "forest edge leaves\n"))
  } else if (the_type == "alvar") {
    phenology_plot <- phenology_plot +
      scale_y_continuous(name = paste0(ylabel, "alvar leaves\n"))
  } else {
    phenology_plot <- phenology_plot +
      scale_y_continuous(name = paste0(ylabel, "moths\n"))
  }
}

stoich_plotting <- all_posteriors %>%
  ungroup() %>%
  select(Component, predictions) %>%
  unnest(predictions) %>%
  mutate(draw = ifelse(Component == "Ratio", draw, draw * 100)) %>%
  group_by(Component, Type) %>%
  nest() %>%
  mutate(phenology_plotting = pmap(
    list(Component, Type, data),
```

```
    phenology_plotting_stoich
  )) %>%
  arrange(Type)

wrap_plots(stoich_plotting$phenology_plotting, ncol = 3, nrow = 3)
```

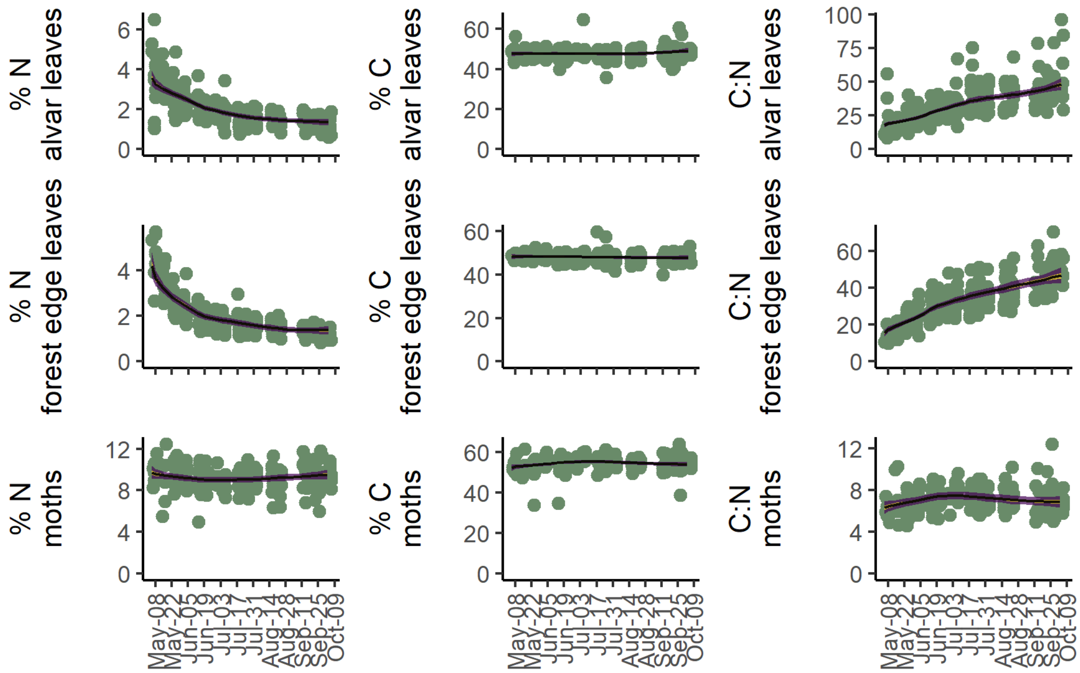

Non-linear seasonal change in C, N, and C:N in alvar and edge leaves and moths.
